# The impact of the Change4Life Food Scanner app on children’s diets and parental psychological outcomes: a randomised pilot and feasibility study

**DOI:** 10.1186/s12889-025-23400-0

**Published:** 2025-07-02

**Authors:** Sundus Mahdi, Jim Chilcott, Nicola J. Buckland

**Affiliations:** 1 Sheffield Centre for Health and Related Research, University of Sheffield, 30 Regent St, Sheffield, S1 4DA UK; 2 Department of Health Sciences, University of York, Heslington, Seebohm Rowntree Building, York, YO10 5DD UK; 3 Department of Psychology, University of Sheffield, ICOSS Building, 219 Portobello, Sheffield, S1 4DP UK

**Keywords:** Mobile applications, MHealth, Digital intervention, App engagement, Childhood obesity prevention, Energy intake, Diet, Sugars, Feasibility study, Behaviour change

## Abstract

**Background:**

The Change4Life Food Scanner app raises awareness of the nutritional content of barcode-scanned packaged food through a variety of visual displays. This study investigated (1) the feasibility and acceptability of evaluating the effectiveness of the Food Scanner app in reducing children’s energy (kcal) and sugar (g) intake over a 3-month period, (2) app engagement and (3) the app’s impact on psychological outcomes.

**Methods:**

Adopting a non-blinded parallel trial design, 126 parents of 4-11 year olds were randomly assigned (1:1) through block randomisation sequences into a 3-month intervention consisting of exposure to the Food Scanner app (version 1.6; [*n* = 62]) or no intervention (*n* = 64). Intervention participants were encouraged to use the app for healthier food choices when shopping. Participants completed baseline and 3-month follow-up (3MFU) measures of child dietary intake, psychological, and health economic outcomes. Dietary intake was also assessed at 1-month. The intervention arm additionally completed fortnightly app engagement measures and all participants provided feasibility feedback at 3MFU. Mixed model Analysis of Variance and independent t-tests of mean differences assessed changes in dietary intake. Descriptive analyses were conducted for all other measures. Ethical approval was obtained by the University of Sheffield Research Ethics Committee (026380).

**Results:**

The study was completed by 64 (51%) of 126 participants (29 [45%] in the intervention group and 35 [55%] in the control group). Most participants (> 80%) found the study acceptable, whilst 68% of intervention participants would recommend the app to others. There was a mean difference in daily energy (kcal) intake of 18 (95% CI: -180; 217) at 3MFU, and a mean difference of 10g in sugar intake (95% CI: -3; 23), between conditions, with a greater reduction within the control condition. Average app engagement declined over the study, from 14.1 min (± 14.7) in week 2 to 6.8 min (± 11.6) in week 12. Minor differences in psychological outcomes were observed between conditions.

**Conclusions:**

Despite high attrition, study procedures were deemed feasible. Low app engagement and usage barriers may have impacted app acceptability and related outcomes. Recommendations are provided for future app development and full-scale trial design.

**Trial registration:**

ISRCTN12169303; 12^th^ May 2025. Retrospectively registered.

**Supplementary Information:**

The online version contains supplementary material available at 10.1186/s12889-025-23400-0.

## Background

In the UK, children are overconsuming saturated fat, sugar and salt compared to recommendations [1]. Sugary soft drinks, cakes, biscuits and breakfast cereals, are causing children to consume almost double their daily sugar limits [2]. In 2018, only 18% of 5–15 year olds consumed the recommended five daily portions of fruit and vegetables [3]. Current guidance suggests that children aged 4–10 years should consume between 1378-1817 cal and 18-24g of free sugars (g) [4]. Unless children’s diets improve, almost 50% of the UK population is predicted to be with obesity by 2060 [5].

Over the past decade, there has been an increased focus on the use of mobile applications to improve dietary intake and prevent weight gain. Studies have highlighted the difficulties in achieving sustained intervention effects [6–8], which may be due to difficulties in maintaining long-term app engagement [7, 9, 10]. Although there is mixed evidence regarding the effectiveness of dietary apps in leading to behavioural changes, evidence has suggested positive changes in psychological predictors of behaviour change [8, 11] and nutrition knowledge [12].

Few trials have investigated the effectiveness of dietary apps in improving child outcomes through parental intervention. The MINISTOP RCT was evaluated to prevent childhood obesity in 4.5 year olds. Parents were encouraged to log their child’s food intake via the app where they could receive feedback, information, advice, and strategies on how to improve dietary behaviours. Significant decreases in sugar sweetened beverage consumption were reported at six months in response to the intervention [6, 13]. Vazquez-Paz and colleagues piloted an app which consisted of food benefits and preparation methods, food diaries, personalised goals and child-focused rewards. Improvements to child dietary intake were reported alongside increases in parents’ knowledge of nutrition guidance [8].

**Fig. 1:**
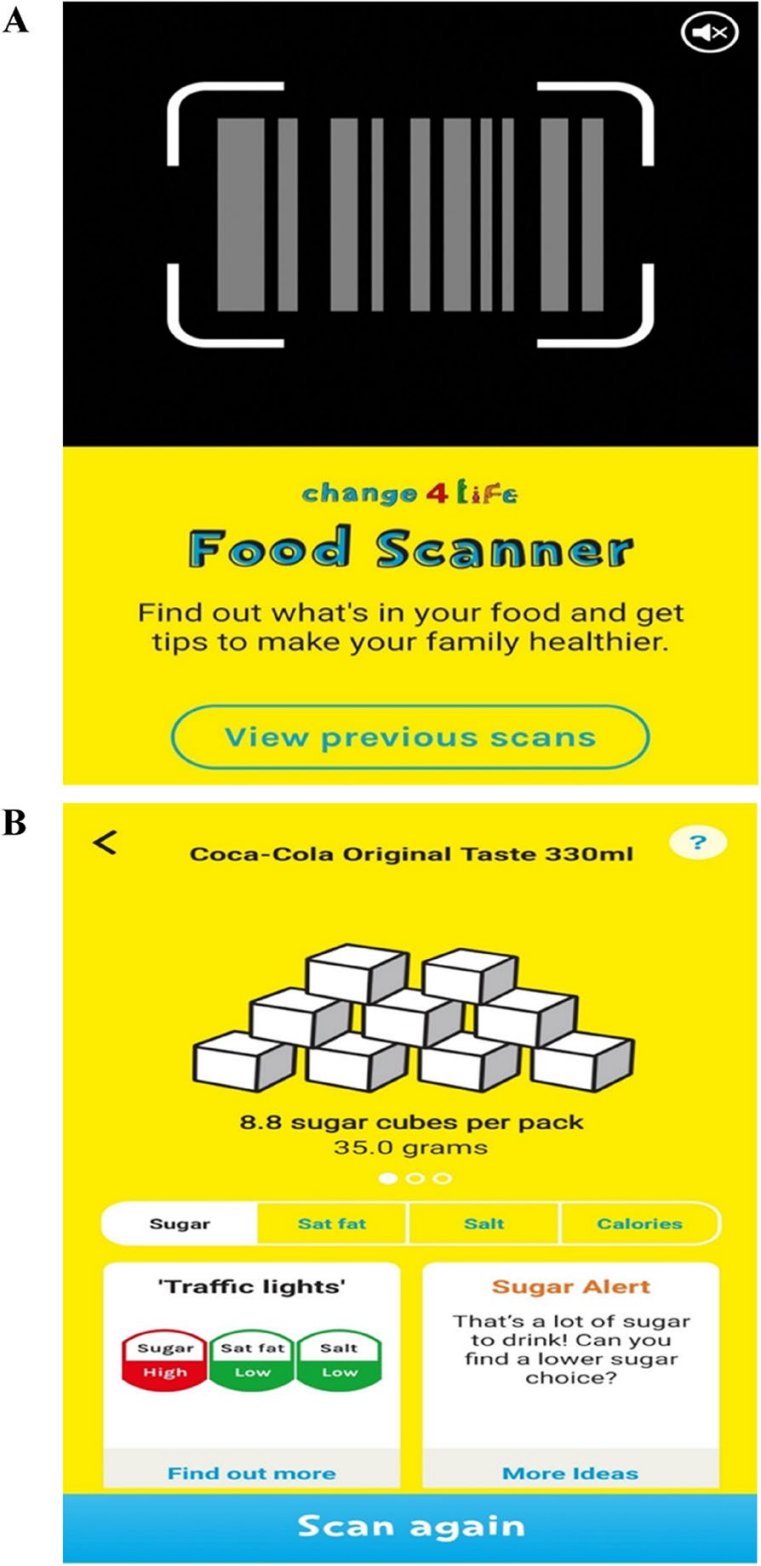
Screenshots of the Food Scanner app (version 1.6). **A** The barcode scanning feature with access to previous scans. **B** Feedback on sugar content through sugar cubes, a traffic light label, and a sugar alert, with an option to scan again

The Change4Life Food Scanner app was designed to support household food purchasing behaviours. The app, originally known as the “Sugar Smart” app, was first released in 2016 by Public Health England as part of a wider campaign [14], that could support pre-existing food policies. The app and campaign aimed to improve children’s diets through raising parental awareness of the fat, sugar and salt content within everyday popular foods. The Food Scanner app provides nutritional feedback on barcode-scanned packaged foods using visual formats like traffic light labels and sugar cube displays (Fig. 1). While nutritional labels can aid healthier choices, their effectiveness is greater among motivated individuals [15, 16]. Visual representations, such as concrete images, enhance understanding and can evoke disgust, leading to positive dietary changes [17–19].

The ‘Sugar Smart’ app was previously evaluated as part of the 2016 Change4Life Sugar Smart campaign [20]. A 2% reduction in sugar intake was observed post-intervention but was not sustained at 12 months. However, compensatory increases in fat and energy intake occurred. Qualitative feedback suggested that (1) the app was useful and fun for child involvement; (2) the use of sugar cubes were an appropriate measure to display information; (3) the app helped food purchasing decisions and (4) prompted discussions around food within households. Limitations included a self-selected, potentially motivated sample, absence of a control group, and lack of app engagement data, making it unclear whether observed effects were due to the campaign or external factors.

The Food Scanner app has undergone a series of major updates to design and content features since its initial release. Mahdi and colleagues investigated behaviour change technique (BCT) content within the Food Scanner app and explored how BCT content evolved with app updates [21]. Findings suggested that the Food Scanner app has the theoretical underpinning of a potentially effective intervention. However, a formal evaluation remains necessary to understand whether app content and related BCTs are sufficient in leading to changes in dietary behaviours.

To our knowledge, no study to date has investigated the impact of the Change4Life Food Scanner app on dietary behaviours through a trial design. Therefore, a pilot randomised controlled trial (RCT) was conducted to test the feasibility and acceptability of evaluating the Change4Life Food Scanner app in reducing children’s sugar and energy intake over a 3-month trial period. An economic component of the evaluation was conducted and reported separately [22]. The primary objective of the current study was to: (1) assess the feasibility and acceptability of evaluating the Food Scanner app; and (2) inform design considerations for a subsequent RCT, such as effect size estimates. Secondary objectives were to: (1) investigate whether there was a reduction in child sugar consumption and overall energy intake between baseline, 1-month and 3-month follow up, between the intervention and control arms; (2) explore parental app engagement over trial duration; and (3) explore differences in parental psychological outcomes between study conditions.

## Methods

### Study design and setting

**Fig. 2:**
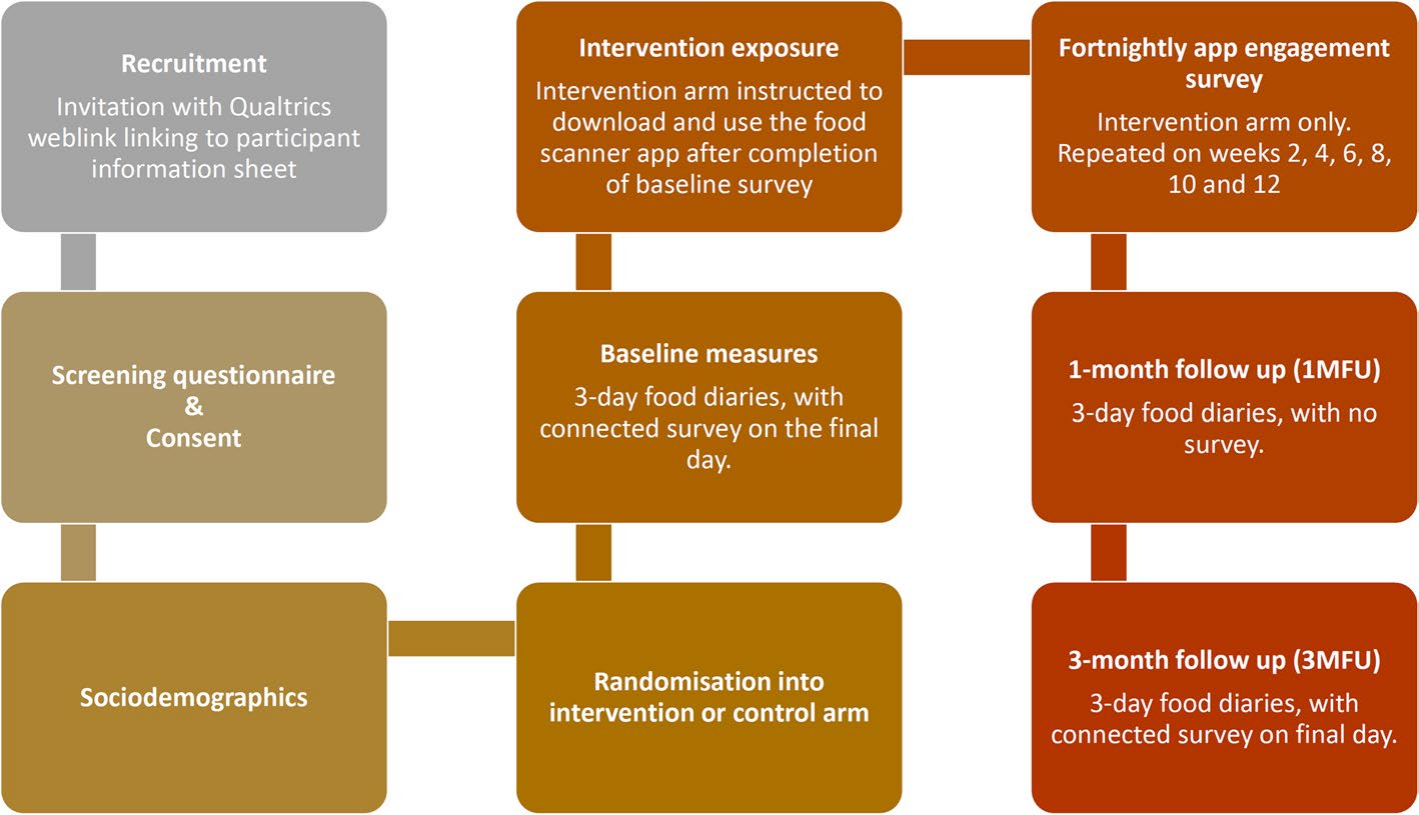
Flowchart of pilot and feasibility study procedure

This was a 3-month non-blinded between-subject online pilot RCT and feasibility study, with 1:1 allocation ratio between intervention and control arms. Parents allocated to the intervention arm were presented with online nutrition guidance, targeting 4–11 year old children, obtained from the Change4Life webpages (now obsolete). This provided a relevant context to then instruct parents to download the Food Scanner app (version 1.6) onto their smartphone, and to use the app to make smarter choices when grocery shopping. Those in the control group did not receive any dietary guidance and were not instructed to download any apps (usual-practice). All participants completed baseline, 1-month follow-up (1MFU) and 3-month follow-up (3MFU) 3-day food diaries, alongside a study survey at baseline and 3MFU. Those in the intervention arm completed fortnightly app engagement measures. A flowchart of the study procedure is presented in Fig. 2.

Recruitment took place between January 2020-June 2020, with a six week pause during March/April 2020 due to COVID-19, focusing on Yorkshire and the Humber, United Kingdom.

The trial was registered on the Open Science Framework (trial registration number: osf.io/62hzt) on 11th March 2020 and the ISRCTN register [ISRCTN12169303] on 12th May 2025 (retrospectively registered). Ethical approval was obtained by the University of Sheffield Research Ethics Committee (026380) in August 2019, and the study was conducted in accordance with the declaration of Helsinki and local governance requirements. Existing literature aided appropriate methods for conducting pilot and feasibility studies [23–25]. The study and reporting of the study adhered to CONSORT for pilot and feasibility studies [26].

### Participant recruitment and randomisation

Although pilot studies do not require sample size calculations [26], they should be large enough to yield useful insights. Viechtbauer and colleagues developed an online calculator to estimate pilot study sample sizes, aiding in identifying trial method issues (e.g., survey question misinterpretation) [27]. With a 95% confidence interval and a 0.05 probability of a problem occurring, the estimated sample size was 58. Accounting for a 20% attrition rate, the target was at least 70 participants.

Participants were recruited using flyers, invitation emails and/or online advertisement posts via primary schools, community centres, social media, online recruitment websites and University of Sheffield mailing lists. Participants in the control condition received a £30 voucher, and those in the intervention arm received a £35 voucher upon study completion. In addition, for every food diary submission, participants were entered into a prize draw for a Virgin Experience Days gift card worth £150.

Participants were presented with a cover story in order to not bias self-reported food intake [28]. Specifically, participants were informed the study was investigating parental attitudes towards dietary online tools. Participants were eligible to take part in the study if they were a parent of a primary school child, aged 4–11 years, lived in Yorkshire and the Humber and were an active grocery shopper or involved in decisions over their child’s food intake. Participants were excluded from the study if they were currently using the Change4Life Food Scanner app or had a child with a health condition with special dietary requirements.

Upon consenting to participate, participants were randomly allocated into a control or intervention arm using a pre-generated randomisation sequence on Microsoft Excel. A randomisation sequence of 50 was produced at first, which was followed by 20 sequences per block thereafter (a total of 4 blocks). The lead researcher (SM) generated the random allocation sequence, enrolled participants and assigned participants to conditions. Researcher blindness to condition allocation was not possible as the distribution of study materials depended on this.

### Measures

Stakeholder engagement with experts in childhood obesity and digital interventions was carried out in November 2019 to advise on study outcomes, study design and evaluation methods [22, 29]. In addition, two patient and public involvement sessions with Parent Governors was conducted contributing to the design of study procedures and materials. Patient and public involvement participants (*n* = 5) were parents of 7–11 year old children, and were diverse in ethnic background. Cognitive debriefing (*n* = 5) was also conducted which involved structured interviews to ensure all study materials, instructions and survey questions were interpreted as intended, in line with research objectives, to increase the study’s content validity [30]. Amendments to study materials were made in accordance with feedback.

#### Sociodemographics

Data on child age and sex, alongside parent ethnic background, educational attainment, household income and number of people living in the household were collected at baseline. Data on household income was used to group participants according to the Index of Multiple Deprivation [31].

#### Study feasibility, acceptability and long term trial engagement

Recruitment and retention rates included numbers who: accessed the participant information sheet (PIS), started completing the consent form, consented, were in the study at 1MFU and 3MFU, completed food diaries (baseline, 1MFU and 3MFU), and completed online surveys (baseline and 3MFU) [32, 33]. Study compliance was assessed by asking participants if they were able to complete all requested study tasks.

Delivery of intervention components was assessed based on the number of participants that downloaded the Food Scanner app, the number of participants who used the app at least once, and number of participants who had previous exposure to the Food Scanner app [34].

Study acceptability was assessed at 3MFU, where participants were asked to feedback on their study experience [32, 34]. Closed-ended questions enquired about the extent to which the study was easy to complete, time consuming/demanding, whether receiving reminders to complete study tasks was helpful, and whether participants completed all requested study tasks. Five response options were provided ranging from high agreeability to low agreeability. Open-ended questions explored barriers to task completion, potential engagement improvements, and additional comments. Participants were also asked, at 3MFU, to rate their level of agreeability ((1) strongly agree – (5) strongly disagree) on whether food diaries affected what their child ate or what they had recorded (adapted from Buckland et al. [35]).

Long-term engagement was assessed by asking participants if they would continue for a 12-month trial ((1) definitely yes - (5) definitely no) [32].

Intervention participants provided feedback on the Food Scanner app's likeability [11], usefulness, and the app’s use of sugar cube images [36]. The study also assessed their ability to make healthy food choices and understand nutritional labels. Open-ended questions gathered qualitative feedback on app likes/dislikes, improvement suggestions for usability and diet support, and barriers to use.

#### Dietary assessment

Three-day food diaries of child food intake were completed by participating parents using myfood24® at baseline, 1MFU and 3MFU. This included two weekdays and one weekend day [37]. Participants were asked to complete all three food diaries over 7 days. Myfood24® is a validated user-friendly online dietary assessment tool [38]. Participants can search for food items for breakfast, lunch, dinner and snacks, and suggestions are also made for commonly missed items. Users can also select portion sizes through pictorial aid and build recipes. Nutrient analysis is undertaken on behalf of the researcher by myfood24® [39].

#### App engagement

Intervention arm participants were asked to report on their app engagement fortnightly through a short online survey. Measures assessed the number of days in which the app was used, and the average time spent using the app, which were used to calculate total app engagement time (minutes). Participants were also asked to report the number of items scanned every two weeks.

#### Predictors of behaviour change

Psychological predictors of behaviour change were assessed at baseline and 3MFU, guided by the COM-B model [40]. This model examines capability (psychological capability, knowledge, physical skills), opportunity (social, physical), and motivation (automatic, reflective) as drivers of behaviour change. Most responses used a 5-point Likert scale, with higher scores indicating greater capability, opportunity, or motivation. Attitudes and perceived control over child sugar intake were also assessed [41]. Survey questions are in Additional File 1.

#### Quality of life, healthcare resource use and productivity

Survey respondents completed a parent-proxy of a short validated paediatric health-related quality of life instrument, the Child Health Utility 9 Dimension scale [42, 43]. Data was captured on healthcare service use in the last 3 months (including number of times and total length of time per contact) [44]; school absenteeism due to a health problem [45]; and workplace absenteeism due to child’s health [46]. These outcomes are analysed separately in an economic evaluation of the Change4Life Food Scanner app [22] and are not reported here.

#### Potential external influencers

##### Physical activity

Self-reported measures of moderate intensity physical activity frequency were collected at baseline [47]. This was defined as, “… causes people to get warmer, breathe harder and their hearts to beat faster” [48]. Participants were asked about the frequency of child moderate intensity physical activity ((1) daily to (5) never). Open-ended responses allowed participants to indicate average time in minutes their child was engaged in moderate intensity physical activity on weekdays and weekends.

##### Awareness of external food policies

Questions enquired about the sugar tax, the use of Change4Life resources, and familiarity with public health campaigns. Five questions measured participant exposure to relevant food policies that may have had an impact on their behaviour during the time of the study at 3MFU. This was asked to provide an understanding of the wider food system and how the external policy context affects parental feeding practices.

##### Impact of the Coronavirus pandemic

In March 2020, 10 weeks into recruitment, the UK entered a national lockdown, prompting 3MFU measures to assess COVID-19's impact on child diet, food choices, parental purchasing behaviour, study participation, and Food Scanner app use (intervention only). Participants rated agreement with statements comparing food purchasing behaviour pre- and post-lockdown. Some questions were adapted from Buckland et al. [49], while others were newly developed.

### Study Procedure

#### Recruitment, participant retention and study withdrawal

Participants were invited via a weblink directing them to an online survey with study details. After consenting, they completed sociodemographic measures and provided contact details before randomisation (but not exposure) to study conditions.

Reminders (up to two per survey or diary entry) were sent via SMS or email, alongside SMS alerts for emailed food diary links. Participants were also contacted midway through the study and one week before their 3-month follow-up.

Non-responsive participants received an email confirming withdrawal and were invited to complete a short online survey (Qualtrics, Provo, UT) on their reasons for withdrawal and suggestions for improved study engagement. Those who responded were entered into a £25 Love2Shop voucher prize draw.

#### Baseline and follow-up measures

Participants received an email invitation to complete three food diaries over seven days, each with a unique link. Parents were encouraged to complete diaries with their child. After submitting the third diary, they were directed to the baseline online survey, which was manually sent to those who missed the deadline.

Intervention-arm participants were then asked to download and use the Food Scanner app (version 1.6), while the control group received no further instructions. Two weeks later, intervention participants reported app engagement via an online survey, repeated fortnightly with email/SMS reminders for non-responders.

Four weeks (1MFU) and 12 weeks (3MFU) after completion of baseline measures, all participants were invited to complete three-day food diaries again. After the 3MFU diaries, they completed a follow-up survey covering baseline measures, food policy awareness, COVID-19 impacts, and study acceptability. Intervention participants also provided feedback on app engagement and acceptability.

### Statistical analyses

Study feasibility was primarily explored through frequency (n[%]) calculations of participant recruitment, retention, food diary and survey completion rates. Frequencies were also investigated for study, food diary and Food Scanner app acceptability measures, in addition to reasons for study withdrawal. Qualitative data was also acquired through open-ended responses providing participant insights into study and intervention feasibility and acceptability, alongside reasons for study withdrawal. Open-ended responses were analysed based on qualitative methods of thematic analysis. Thematic analysis provides the researcher with the main themes, or patterns, emerging from responses, organised hierarchically. Utilising a grounded theory approach, codes were derived based on what emerged from responses, which were then grouped into themes [50]. Themes are presented alongside supporting quotes, and data was additionally presented and discussed in the context of understanding quantitative outcomes.

Average energy (kcal) and sugar (g) intake of completed food diaries were calculated for baseline, 1MFU and 3MFU. In the case where participants did not complete all 3 food diaries, an average was calculated based on the number of food diaries completed. Skewness and Kurtosis tests were undertaken to check for normality, alongside z-scores (results not reported). Extreme data points (i.e. outliers) were removed ahead of analysis in instances where z-scores were above 3 standard deviations [51]. A mixed design Analysis of Variance explored the preliminary efficacy of the Food Scanner app on energy (kcal) and sugar (g) intake, and to obtain effect size estimates, at 1MFU and 3MFU. As the study was not powered to detect significant differences, no covariates were imputed into the model. Mean differences (95% CI) in dietary intake between baseline and 1MFU/3MFU within participants were calculated. Mean differences between intervention and control arms were also explored using independent samples t-tests. Each analysis was based on complete cases. Statistical analysis was conducted on IBM SPSS Statistics 15.

App engagement was calculated for weeks 2, 4, 6, 8, 10, and 12 of the intervention based on (1) average (mean [± SD]) number of times the app was used (i.e. frequency of use); (2) average (mean [± SD]) time spent (minutes) using the app at any one time (i.e. duration of use); and (3) the average (mean [± SD]) of the total time spent (minutes) using the app, as a calculation of frequency and duration in weeks. The mean [± SD] of participant reported scanned items were also calculated at 2, 4, 6, 8, 10, and 12 weeks. Results were plotted onto a line graph depicting app use over time.

Frequencies [%]) were explored on behaviour change measures and compared between control and intervention conditions, and between baseline and 3MFU. A within-subjects comparison of psychological predictors of behaviour change was conducted (Mean [± SD], alongside paired samples t-tests to explore differences (95% CI) between baseline and 3MFU.

Response frequencies (n[%]) were calculated to assess the impact of COVID-19 and other external factors. Mean [± SD] of average child weekday and weekend physical activity was additionally explored.

Removal of outliers was applied for dietary, physical activity and app engagement data, and did not exceed more than 2 exclusions per variable. Data from 5-point Likert scales were transformed (and reverse coded where necessary) into 3-point scales representing low, medium and high agreeability outcomes.

Given this is a pilot and feasibility study, statistical analysis explored mean differences and confidence intervals. Preliminary inferential statistics were conducted at the 5% significance level for exploratory purposes only [52], whilst acknowledging that hypothesis testing is a contentious issue within the reporting of pilot and feasibility studies as they are usually underpowered to detect statistical significance [24]. However, this is a commonly adopted method by researchers [52].

## Results

### Participant recruitment, retention and compliance

In total, 201 potential participants accessed the survey webpage to the participant information sheet. Of these, 25 did not provide consent. As such, 176 were assessed for eligibility whereby 4 declined to participate, 20 did not pass the eligibility criteria, and 26 did not provide an email address. The remaining sample consisted of 126 parents, of which 62 were allocated to the intervention arm and 64 to the control arm. Participants were recruited via Facebook (*n* = 54; 42.9%), University of Sheffield mailing list (*n* = 23, 18.3%) and via their family and friends (*n* = 15, 11.9%). The remainder of participants did not provide any information (*n* = 34, 27%).

**Fig. 3:**
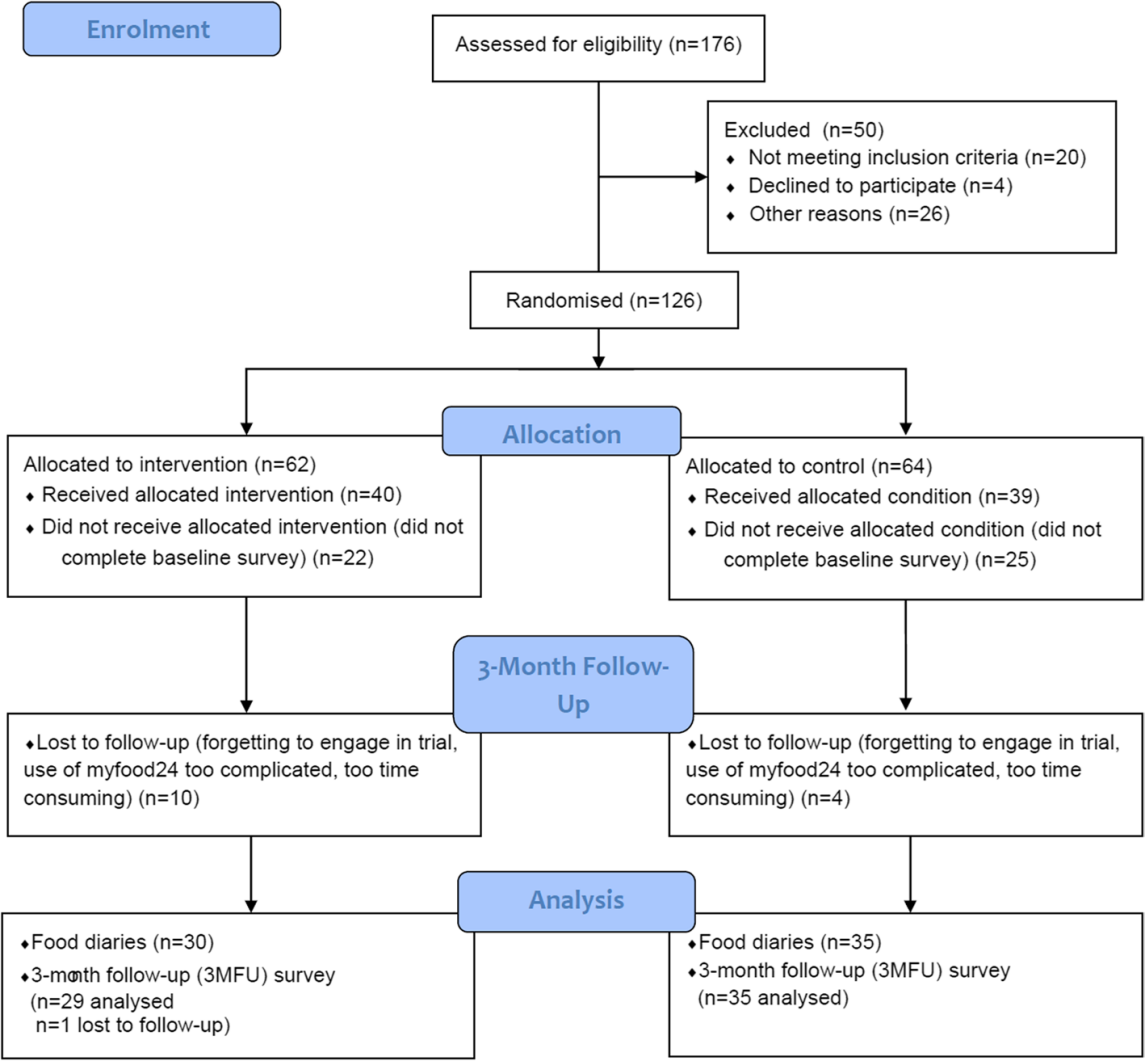
CONSORT flow chart for the Change4Life Food Scanner app pilot and feasibility trial

In terms of study compliance, the first baseline food diary was completed by 87 of 126 (69%) parents (control: *n* = 43; intervention: *n* = 44). As such, 39 parents did not complete the first food diary, and were considered dropouts from the beginning. All 3 food diaries were completed by 77 of 126 (61%) participants at baseline (control: *n* = 38; intervention: *n* = 39), 51 (40%) participants at 1MFU (control: *n* = 32; intervention: *n* = 19; 61 [48%] completed at least one food diary) and 52 (41%) participants at 3MFU (control: *n* = 30; intervention: *n* = 22; 66 [52%] completed at least one food diary). All 3-day food diaries at baseline, 1MFU and 3MFU were completed by 25 control and 16 intervention participants. The baseline survey was completed by 79 of 126 (63%) parents, and therefore 39 participants received the allocated control condition, and 40 participants received the allocated intervention condition. Finally, 65 of 126 (52%) participants completed at least one baseline and one 3MFU food diary (control: *n* = 35; intervention: *n* = 30), whilst 64 of 126 (51%) participants completed the final 3MFU survey (control: *n* = 35; intervention: *n* = 29), and 62 (49%; control: *n* = 29; intervention: *n* = 33) dropped out. Out of the 40 participants that were exposed to the intervention, 6 (15%) did not download the Food Scanner app. For the CONSORT flowchart, see Fig. 3.

### Sociodemographics

Amongst study completers (*n* = 64), the mean age of the children was 6.9 ± 2.2 years. There was 55% male children within the intervention arm and 36% in the control. Approximately 80% of the parental sample was Caucasian, of which 71% had completed higher education, and approximately half were from the two least deprived income quintiles in both study conditions. Meanwhile, 24% of parents in the intervention arm were in the two most deprived income quintiles in comparison to 17% in the control arm. Table 1 outlines the distribution of demographics at baseline of all participants and of study completers within the intervention and control arms. Additional File 2 explores participant characteristics of study dropouts. There were no differences in sociodemographics between completers and dropouts, except for ethnicity; a greater number of Asian participants dropped out of the study (15%) than completed it (5%).

**Table 1:** Demographics of study participants

		All participants			Study completers^a^		
		Total *n* = 126	Intervention^b^ *n* = 62	Control^c^ *n* = 64	Total *n* = 64	Intervention *n* = 29	Control^d^ *n* = 35
Child age (years)	Mean (±SD)	6.81 (2.04)	6.77 (1.77)	6.85 (2.28)	6.9 (±2.2)	6.8 (±2.0)	7.1 (±2.4)
Child sex	N (%) Female	60 (52)	26 (46)	34 (57)	34 (55)	13 (45)	21 (64)
	N (%) Male	56 (48)	30 (54)	26 (43)	28 (45)	16 (55)	12 (36)
Parent Ethnicity	N (%) White British	81 (71)	41 (75)	40 (68)	42 (68)	20 (69)	22 (67)
	N (%) White other	9 (8)	5 (9)	4 (7)	6 (10)	4 (14)	2 (6)
	N (%) Asian	11 (10)	4 (7)	7 (12)	3 (5)	2 (7)	1 (3)
	N (%) Mixed White and Black	4 (4)	3 (6)	1 (2)	3 (5)	2 (7)	1 (3)
	N (%) Other	9 (8)	2 (4)	7 (12)	8 (13)	1 (3)	7 (21)
Parent Education	N (%) Higher education^e^	79 (69)	39 (71)	40 (68)	44 (71)	21 (72)	23 (70)
	N (%) Other	35 (31)	16 (29)	19 (32)	18 (29)	8 (28)	10 (30)
Household Income (quintiles)	N (%) Q1 – most deprived	16 (13)	10 (16)	6 (9)	8 (13)	5 (17)	3 (9)
	N (%) Q2	5 (4)	2 (3)	3 (5)	5 (8)	2 (7)	3 (9)
	N (%) Q3	16 (13)	6 (10)	10 (16)	7 (11)	3 (10)	4 (11)
	N (%) Q4	28 (22)	14 (23)	14 (22)	13 (20)	5 (17)	8 (23)
	N (%) Q5 – least deprived	40 (32)	18 (29)	22 (34)	23 (36)	11 (38)	12 (34)
	N (%) Unknown	21 (17)	12 (19)	9 (14)	8 (13)	3 (10)	5 (14)

N.B. Percentages rounded up to 0 decimal places

^a^Study completers defined as those who have completed the study - completing both baseline and 3MFU surveys

^b^Six missing cases for age and sex; 1 additional missing case for all other variables

^c^Four missing cases for age and sex; 1 additional missing case for all other variables

^d^Two missing cases for gender, education and ethnicity

^e^Defined as higher education qualification below degree level, degree level qualification, or a Masters/PhD or equivalent

### Feasibility, acceptability and long-term trial engagement

Table 2 presents feasibility and acceptability results. Among study completers, 48 of 64 (76%) completed all tasks. Reported barriers to task completion included time constraints and forgetfulness (Table 3, Section A). However, only 10 of 64 (16%) found the study too time-consuming.

**Table 2:** Feasibility and acceptability of study procedures by study completers, n (%)

Measure	n	High agreeability	Medium agreeability	Low agreeability
*Study procedures*				
Study was easy to complete^a^	64	51 (80)	8 (13)	5 (8)
Participating in the study was time consuming/demanding^b^	64	10 (16)	23 (36)	31 (48)
Receiving reminders to complete food diaries and surveys was helpful^c^	64	62 (97)	2 (3)	0 (0)
All requested study tasks were completed^d^	63	48 (76)	13 (21)	2 (3)
*Food diaries*^c^				
I did not report everything my child ate	63	1 (2)	7 (11)	55 (87)
I changed what my child actually ate to make it easier to record	64	6 (9)	5 (8)	53 (83)
It had no effect on what my child ate	64	44 (69)	11 (17)	9 (14)
It was easy to use	64	44 (69)	11 (17)	9 (14)
I found it too much work	64	17 (27)	15 (23)	32 (50)
*Sustainability*				
Willing to continue with study for 9 more months if the study was extended to a 12-month follow-up^e^	62	45 (73)	11 (18)	6 (10)

^a^Original response options were: extremely easy, somewhat easy, neither easy nor difficult, somewhat difficult, extremely difficult

^b^Original response options were: a great deal, a lot, a moderate amount, a little, none at all

^c^Original response options were: strongly agree, somewhat agree, neither agree nor disagree, somewhat disagree, strongly disagree

^d^Original response options were: completed all tasks, completed the majority of the tasks, completed a fair amount of the tasks, completed very few of the tasks

^e^Original response options were: definitely yes, probably yes, might or might not, probably not, definitely not

**Table 3:** Open-ended survey questions, themes and supporting statements

Theme and codes	Example Quotes
SECTION A:* What prevented you from completing all study tasks?*	
Forgetfulness	“Was finding the time and not forgetting.”
Time	“Time consuming with COVID as went back to work and shopping was a rush and didn’t allow me extensive time to scan food and use the app or fill in diaries.” “I am a busy NHS worker who has worked more over the previous few months due to the Covid pandemic.”
SECTION B:* Was there anything we could have done to keep you more engaged in completing food diaries and surveys throughout this study?*	
Food diary completion	“Being able to complete the food diaries retrospectively would have been helpful.” “I could have done with receiving the food diary email on a Monday rather than mid week when half the week was already gone. The layout of the food diary was not very user friendly. I found it hard to use on my phone.”
myfood24® improvements	“Perhaps when a diary is partially completed but not yet submitted a reminder to ask you to submit would have been useful.” “My son has a plant-based diet and it was often very difficult to find the exact things that he eats. We also usually cook most from scratch and do not eat a lot of processed food, but it was sometimes impossible to find something like'red onion', whereas the list with red onion in processed food was very long.” “The food diary does not include all food we had (in terms of brand, cooking methods, ingredients etc).”
Task for the child	“Maybe have something for the child themselves to do.”
Greater monetary incentive	“I’d happily continue with the study subject to reward.” “£30 seems a bit low in hindsight for the participation and time committed.”
SECTION C:* Food Scanner app acceptability*	
App usefulness ● Helpful feedback of nutritional information ● No added value ● Assumes lack of nutrition knowledge ● App assumes ample time to scan products ● App does not consider meal prepping and shopping list ● App only useful before changes made ● Don’t need to continuously use app ● App isn’t useful for personal grocery choices ● Not useful for those that cook ● Limited usefulness and information provision ● Not useful for those providing balanced diet ● Does not address fussy eaters ● Does not consider other important macronutrients ● Limit to app use ● Don’t need to use it	“Easy to use and understand broke down nutritional labels into comprehensible information allowing informed and healthy decisions.” “The app assumes that you don't know much about child nutrition in the first place. As a parent I regularly meal plan and write a shopping list I don't just wander round the supermarket scanning random items. The app also assumes you have ample time to wander round when in reality I like to spend the least amount of time shopping.” “Not sure. Once you know the content of a product you don’t need to scan it again. It was very useful at first but once we’d made changes we didn’t need it as much.” “I think it is aimed at parents who only buy ready made food for their children. It is not helpful for parents who cook from scratch. It also assumes that you know very little about basic nutrition. For example I know a can of Coke is unhealthy and contains several cubes of sugar, I don't need an app to tell me. I wouldn't bother to scan several to see which had the least amount of sugar, I just wouldn't buy it in the first place. I didn't use the app after a while as it didn't give me any further information.” “Too time consuming, just as easy to look at a label.”
Better product recognition ● Not everything scanned recognised ● Embed search features ● Section on popular scanned items	“Better range of goods recognised.” “Maybe search for an item rather than having to scan.” “Include more items.” “It didn't always recognise the items I scanned. I shop at aldi and a lot of the products weren't on there. It only seemed to recognise branded products. Once you scanned an item it wasn't very easy to find your way round the app to things such as recipes.”
Information provision and monitoring ● Resources ● Display information by serving ● Improve display of information presented ● Make information attached to food labels ● Health behaviour progress chart ● Score items scanned ● Swap ideas ● Recipe ideas	“I liked the link to the change to life website for the NHS recipes.” “Have the amount per serving.” “Don't use the app, make it attached to the food label.” “Examples of healthy treats advertised on it.” “A chart to show positive changes to see progress.” “The information per portion clearer.” “Recipe ideas? Like alternatives for birthday party treats that have less sugar in?”
Presentation ● Aesthetically pleasing ● Tone/Preachy ● Simple colours ● Colour clashes impact concentration ● Simple encouraging terms	“It was very easy to scan products and see their information. It was bright and interested my daughter too.” “Simple encouraging terms.” “Less colour clashes makes it hard to concentrate.” “Simpler colours.”
Rewards and incentives ● Money off vouchers ● Rewards system ● Access to incentives for healthier products	“Incentives- money off vouchers, rewards system” “Possibly incentives for parents that otherwise may choose cheaper options like potential discount and money accumulators.” “Give free healthy food for using the app.”
Child involvement ● Chart to log child’s progress ● More child-friendly ● Gamification	“Engage children directly to integrate with daily life.” “Maybe a chart to log a child’s progress when they’ve made swaps.” “make into a game to get children involved in making food choices.”
Personalisation ● Individual targets ● Link with social media	“Provide individual targets.” “Maybe link with social media.”
Convenience and practicality ● Ease and speed of use ● Fun ● Inconvenient ● Use less phone memory ● Time consuming ● Daily reminders ● Forgot ● COVID-19 impacting diets ● COVID-19 impacting use	“It was easy to use and handy to have on my mobile so when I was in a shop I could use it to decide which was a healthier choice of product.” “It consumes my phone memory.” “Too time consuming.” “It’s difficult to get the app out in shops and start scanning everything before making a purchase.” “I have to prepare food quickly so didn’t have time.” “Daily reminders to use it.” “I often forgot about the app.” “During COVID-19 I don’t really like getting my phone out in supermarkets, especially without disinfecting first.” “It was easy to use, very user friendly.”

Food diary acceptability was assessed. Of 64 respondents, 44 (69%) found myfood24® easy to use, while 17 (27%) felt it was too much work, citing issues like limited food options and mobile usability. Most (*n* = 55, 87%) disagreed with withholding dietary information from food diaries, and 53 (83%) denied altering their child’s diet for easier recording. Additionally, 44 (69%) agreed that food diaries did not influence their child’s eating habits.

Study acceptability was investigated at 3MFU (see Table 2). In general, the study was considered acceptable. Amongst study completers, 51 (80%) felt that the study was easy to complete, and 62 (97%) felt that task completion reminders were helpful. If the study was extended to 12 months, 45 of 62 (73%) participants were willing to continue for another 9 months (see Table 2).

Participant engagement strategies were explored (Table 3, Section B), revealing four key themes: (1) Food diary flexibility – some participants preferred choosing their diary completion day (i.e. retrospective completion) rather than recording in real-time; (2) Myfood24® improvements – a few participants suggested enhancing the database and adding diary submission reminders; (3) Child involvement – some participants recommended incorporating child-focused tasks to increase study engagement; (4) Monetary incentive – some participants felt compensation was insufficient, while others indicated a willingness to continue participating subject to the incentive provided.

### Intervention acceptability

Intervention participants rated the Food Scanner app's acceptability. While 25 of 28 (89%) found sugar cube images easy to understand, only 16 (57%) found them useful. Additionally, 24 (86%) supported adding these images to food labels. Despite positive feedback, 20 (71%) said the app did not improve their food purchasing behaviour.

When asked to feedback on their likeability of the Food Scanner app, 16 of 28 (57%) participants thought the app was helpful, 24 (86%) thought the app was easy to use, 14 (50%) enjoyed using the app, 17 (61%) liked the app, and 18 (64%) said that they would recommend the app to others.

When asked about the Food Scanner app's effectiveness, 16 of 28 (57%) reported it helped reduce children’s high-sugar snack consumption and increased ability to make healthier food choices for their child. However, open-ended responses noted barriers like less healthy diets from grandparents and COVID-19 placing difficulty on dietary behaviour changes. One respondent noted that the survey did not ask about dietary changes made after using the app.

Open-ended questions gathered feedback from intervention participants on their experiences with the Food Scanner app, barriers to engagement, and improvement suggestions (Table 3, Section C). Eight key themes emerged. (1) App usefulness – some found the app helpful for nutritional feedback, while others felt it was unsuitable for home cooking, provided basic nutrition information, and was redundant for those using front-of-pack labels. (2) Better product recognition – participants suggested improving the barcode scanner to recognise more items, adding a product search feature, and storing frequently scanned items. (3) Information provision and monitoring – participants recommended enhancements to nutritional information displayed (e.g., per serving), low-sugar swaps, and behaviour tracking. One participant appreciated links to external dietary resources. (4) Presentation – while most liked the app’s design and found it aesthetically pleasing, two disliked the colour-scheme and a minority wanted more encouraging language. (5) Rewards and incentives – several parents reported that incentives, such as reward systems, prize incentives, and discount vouchers, would boost app engagement. (6) Personalisation – app improvement proposals included adding individual targets, social media sharing, and child-friendly features to engage children directly. (7) Promote child involvement – some participants suggested the app should be more child-friendly and engage children directly. (8) Convenience and practicality – while several participants found the app quick and fun, others saw it as inconvenient in supermarkets, especially during COVID-19. Some found it time-consuming, forgot to use it due to busy schedules, or felt it consumed too much phone memory. In-app reminders were suggested to prompt use.

### Study withdrawal

Of 62 dropouts, 6 completed the withdrawal survey. Reasons included myfood24® being too complicated, study tasks being too time-consuming, forgetting to complete diaries, insufficient compensation, and technical issues. Open-ended feedback noted challenges in logging vegan diets and finding exact foods, especially for recipes.

### Preliminary effects of the intervention

#### Dietary assessment

Energy (kcal) and sugar intake (g) outcomes within- and between groups are reported in Table 4, based on paired-samples data from 1 and 3MFU. At 1MFU, baseline energy intake was 1772.7 (± 404.8) kcal in the intervention arm, and 1568.1 (± 459.2) kcal in the control arm. Energy intake reduced by −102 kcal (95% CI: −285; 80) in the intervention group, and −1856 (95% CI: −308; −64) in the control group. At 3MFU, baseline intake was 1763.2 (± 421.8) kcal in the intervention arm, and 1552.1 (± 470.8) kcal in the control arm. Energy intake reduced by −157 (95% CI: −301; −13) in the intervention group, and −175 (95% CI: −316; −34) in the control group. Mean differences in energy intake were non-significant at both 1MFU (83 kcal, 95% CI: −1223; 289) and 3MFU (18 kcal, 95% CI: −180; 217) between the intervention and control conditions, with a greater reduction in the control condition.

**Table 4:** Mean differences (± SD) in energy (kcal) and sugar (g) intake between baseline and follow-up

Outcome	Intervention			Control^a^			Total mean difference (95% CI)
	Baseline	1 month	Difference (95% CI)^b^	Baseline	1 month	Difference (95% CI)	
Energy (kcal)	1773 (±405)	1670 (±338)	−102 (−285; 80)	1568 (±459)	1382 (±330)	−186 (−3078; −64)	83 (−123; 289)
Sugar (g)	77.1 (±21.5)	78.4 (±33.4)	1.4 (−12.6; 15.4)	76.2 (±24.1)	67.3 (±25.6)	−8.9 (−14.8; −2.9)	10.2 (−3.0; 23.5)
	Baseline	3 months	Difference^c^	Baseline	3 months	Difference	
Energy (kcal)	1763 (±422)	1606 (±446)	−157 (−301; −12)	1552 (±471)	1377 (±278)	−175 (−316; −34)	18 (−180; 217)
Sugar (g)	80.1 (±25.8)	78.9 (±33.0)	−1.3 (−12.8; 10.2)	75.5 (±23.4)	64.3 (±25.9)	−11.2 (−18.4; −3.9)	9.9 (−2.9; 22.7)

N.B. Data is based on complete case analysis, after the removal of outliers 3 standard deviations from the mean

^a^Energy, *n*= 35; sugars, *n*=25 for both 1-month and 3-month follow-up

^b^Energy, *n*= 25; sugars, *n*=24

^c^Energy, *n*= 30; sugars, *n*=28

Baseline sugar intake, for the 1MFU analysis, was 77.1 (± 21.5) in the intervention arm, and 76.2 (± 24.1) in the control arm. Sugar intake reduced by 1.4 (95% CI: −12.6; 15.4) in the intervention group, and −8.9 (95% CI: −14.8; −2.9) in the control group. At 3MFU, baseline intake was 80.1 (± 25.8) in the intervention arm and 75.5 (± 23.4) in the control arm. Sugar intake reduced by −1.3 (95% CI: −12.8; 10.2) in the intervention group, and −11.2 (95% CI: −18.5; −3.9) in the control group. Mean differences in sugar intake were non-significant at both 1MFU (10 g, 95% CI: −3; 23) and 3MFU (10 g, 95% CI: −3; 23) between conditions, with a greater reduction in the control condition.

A 2 × 2 mixed model ANOVA was conducted, with study condition as a between-subjects factor (intervention vs. control) and time as a within-subjects factor (baseline vs. 1MFU and 3MFU). The analysis revealed a within-subjects main effect of time on energy intake at 1MFU, *F*(1, 58) = 7.827, *p* = 0.007, η_p_^2^ = 0.119, and at 3MFU, *F*(1, 63) = 11.204, *p* < 0.001, η_p_^2^ = 0.151, suggesting that irrespective of study condition, energy intake was significantly greater at baseline than follow-up. The analysis also revealed a between-subjects main effect of condition at 1MFU, *F*(1, 58) = 7.860, *p* = 0.007, η_p_^2^ = 0.119, and 3MFU, *F*(1, 63) = 6.143, *p* < = 0.016, η_p_^2^ = 0.089, whereby the intervention arm consumed more calories than the control arm irrespective of time. No interaction between condition and time on energy intake was found at 1MFU, *F*(1, 58) = 0.654, *p* = 0.422, η_p_^2^ = 0.011, or 3MFU, *F*(1, 63) = 0.034, *p* = 0.855, η_p_^2^ = 0.001.

The analysis also revealed no within-subjects main effect of time on sugar intake at 1MFU, *F*(1, 57) = 1.275, *p* = 0.264, η_p_^2^ = 0.022, and 3MFU, *F*(1, 61) = 3.760, *p* = 0.057, η_p_^2^ = 0.058. There was also no between-subjects main effect of condition at 1MFU, *F*(1, 57) = 0.963, *p* = 0.331, η_p_^2^ = 0.017, and 3MFU, *F*(1, 61) = 2.523, *p* = 0.117, η_p_^2^ = 0.04. Finally, there was no interaction between condition and time on sugar intake at 1MFU, *F*(1, 57) = 2.383, *p* = 0.128, η_p_^2^ = 0.040, and 3MFU, *F*(1, 61) = 2.380, *p* = 0.128, η_p_^2^ = 0.038.

#### App engagement

**Fig. 4:**
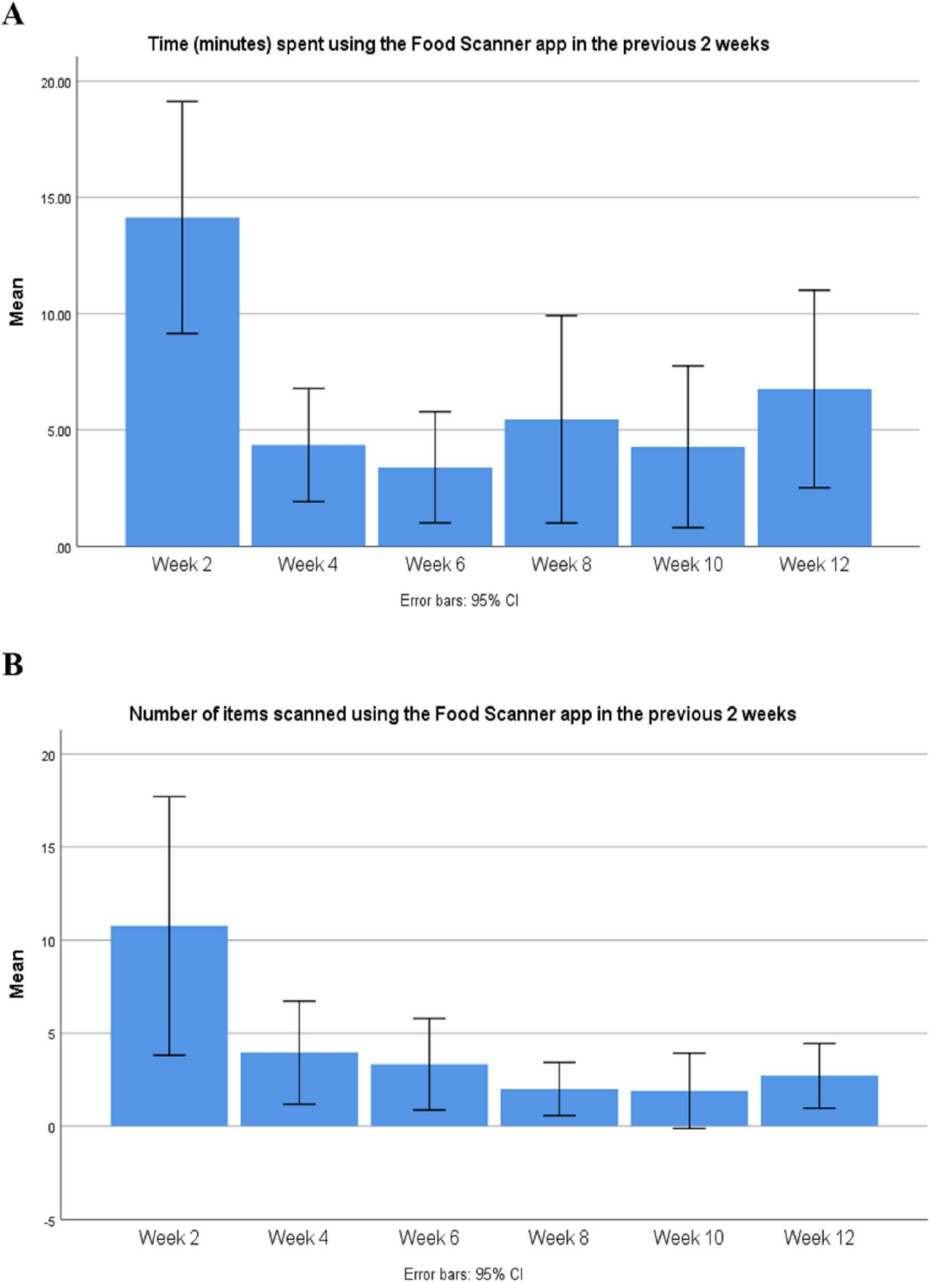
Self-reported Food Scanner app engagement over the 12-week trial period (*n* = 34). **A** Time (minutes) spent using the Food Scanner app in the previous 2 weeks. **B** Number of items scanned using the Food Scanner app in the previous 2 weeks

Results indicated that average app engagement (minutes) decreased over time. During the first two weeks of exposure to the Food Scanner app, participants (*n* = 34) reported an average engagement time of 14.1 min (± 14.7) per two weeks. At 12 weeks, participants (*n* = 29) reported approximately 6.8 min (± 11.6) of app engagement in the previous two weeks (see Fig. 4, panel A). Number of items scanned fortnightly suggested a gradual decrease in app engagement over the trial period (see Fig. 4, panel B). Participants reported an average of 11 scanned items (± 20.5) during the first 2 weeks of app exposure (week 2), and 3 scanned items (± 4.6) in the final 2 weeks of the trial (week 12).

#### Predictors of behaviour change

Predictors of behaviour change at baseline and 3MFU are in Table 5. In the intervention group, the ability to make healthy food choices (psychological capability) dropped from 70% at baseline to 41% at 3MFU, despite improved ease in understanding labels (40% struggled at baseline vs. 28% at 3MFU) and greater tracking of child sugar intake (physical capability; low tracking: 23% at baseline vs. 7% at 3MFU). In the control group, psychological capability remained stable, but label comprehension worsened (39% struggled at baseline vs. 60% at 3MFU), while low nutritional tracking decreased (29% at baseline vs. 17% at 3MFU). Knowledge of children's recommended daily sugar intake remained low, with correct responses increasing from 3 (10%) to 5 (17%) out of 29 in the intervention group and from 6 (19%) to 8 (25%) out of 32 in the control group.

**Table 5:** Comparison of psychological predictors of behaviour change between intervention and control arms, n (%)

Question	Baseline agreeability			3-month follow-up agreeability		
	High	Medium	Low	High	Medium	Low
*Attitudes*						
* How important is it for you that your family eat a healthy diet?*^a^						
Intervention (base, *n*=40; 3MFU, *n*=29)	35 (88)	5 (13)	0	24 (83)	5 (17)	0
Control (base, *n*=39; 3MFU, *n*=35)	33 (85)	6 (15)	0	30 (86)	5 (14)	0
* Having too much sugar leads to disease*^b^						
Intervention (base, *n*=40; 3MFU, *n*=29)	33 (83)	6 (15)	1 (3)	27 (93)	2 (7)	0
Control (base, *n*=39; 3MFU, *n*=35)	36 (92)	3 (8)	0	33 (94)	1 (3)	1 (3)
* When buying food, snacks or drinks for my child, it is important to pay attention to the amount of sugar it contains*^b^						
Intervention (base, *n*=40; 3MFU, *n*=29)	31 (78)	7 (18)	2 (5)	25 (86)	3 (10)	1 (3)
Control (base, *n*=39; 3MFU, *n*=35)	35 (90)	4 (10)	0	34 (97)	1 (3)	0
* For my child to be healthy, I need to be careful how much saturated fat my child eats*^b^						
Intervention (base, *n*=40; 3MFU, *n*=29)	34 (85)	4 (10)	2 (5)	22 (76)	6 (21)	1 (3)
Control (base, *n*=39; 3MFU, *n*=35)	30 (77)	8 (21)	1 (3)	29 (83)	5 (14)	1 (3)
* For my child to be healthy, I need to be careful how much sugar my child eats*^b^						
Intervention (base, *n*=40; 3MFU, *n*=29)	40 (100)	0	0	27 (93)	1 (3)	1 (3)
Control (base, *n*=39; 3MFU, *n*=35)	39 (100)	0	0	34 (97)	1 (3)	0
* For my child to be healthy, I need to be careful how many calories my child eats*^b^						
Intervention (base, *n*=40; 3MFU, *n*=29)	17 (43)	18 (45)	5 (13)	10 (35)	9 (31)	10 (34)
Control (base, *n*=39; 3MFU, *n*=34)	17 (44)	9 (23)	13 (33)	20 (59)	6 (18)	8 (24)
*Perceived behavioural control*						
* How much control do you have over your child’s sugar consumption?*^c^						
Intervention (base, *n*=40; 3MFU, *n*=28)	31 (78)	9 (23)	0	19 (68)	9 (32)	0
Control (base, *n*=39; 3MFU, *n*=35)	33 (85)	6 (15)	0	24 (69)	11 (31)	0
*COM-B measures: Physical capability*						
* How often, if at all, do you keep track of how much sugar your child eats or drinks each day?*^d^						
Intervention (base, *n*=40; 3MFU, *n*=29)	15 (38)	16 (40)	9 (23)	10 (35)	17 (59)	2 (7)
Control (base, *n*=38; 3MFU, *n*=35)	15 (40)	12 (32)	11 (29)	18 (51)	11 (31)	6 (17)
*COM-B measures: Psychological capability*						
* How easy or difficult do you find it to limit your child’s sugar intake to the amounts recommended in the above guidelines?*^e^						
Intervention (base, *n*=40; 3MFU, *n*=29)	9 (23)	7 (18)	24 (60)	7 (24)	3 (10)	19 (66)
Control (base, *n*=38; 3MFU, *n*=35)	14 (37)	6 (16)	18 (47)	14 (40)	4 (11)	17 (49)
* How much do you think you know about making healthy food choices?*^f†^						
Intervention (base, *n*=40; 3MFU, *n*=29)	28 (70)	12 (30)	0	12 (41)	16 (55)	1 (3)
Control (base, *n*=39; 3MFU, *n*=35)	26 (67)	13 (33)	0	22 (63)	13 (37)	0
* “Too much sugar intake for my child increases their risk of obesity”*^b^						
Intervention (base, *n*=40; 3MFU, *n*=29)	39 (98)	1 (3)	0	29 (100)	0	0
Control (base, *n*=39; 3MFU, *n*=35)	39 (100)	0	0	34 (97)	1 (3)	0
“*Nutritional labels are hard to understand”*^b†^						
Intervention (base, *n*=40; 3MFU, *n*=29)	16 (40)	10 (25)	14 (35)	8 (28)	8 (28)	13 (45)
Control (base, *n*=39; 3MFU, *n*=35)	15 (39)	11 (28)	13 (33)	21 (60)	6 (17)	8 (23)
*COM-B measures: Automatic motivation*						
* How concerned, if at all, are you about your child consuming more sugar than what is recommended?*^g^						
Intervention (base, *n*=40; 3MFU, *n*=29)	10 (25)	28 (70)	2 (5)	8 (28)	19 (66)	2 (7)
Control (base, *n*=38; 3MFU, *n*=35)	10 (26)	22 (58)	6 (16)	10 (29)	17 (49)	8 (23)
* To what extent do you want to keep your child’s sugar consumption within recommended guidelines?*^g^						
Intervention (base, *n*=40; 3MFU, *n*=29)	20 (50)	19 (48)	1 (3)	18 (62)	10 (34)	1 (3)
Control (base, *n*=38; 3MFU, *n*=35)	29 (76)	9 (24)	0	26 (74)	9 (26)	0
*COM-B measures: Reflective motivation*						
* To what extent do you intend to keep your child’s sugar consumption within recommended guidelines?*^h^						
Intervention (base, *n*=40; 3MFU, *n*=29)	32 (80)	7 (18)	1 (3)	20 (69)	8 (28)	1 (3)
Control (base, *n*=38; 3MFU, *n*=35)	34 (89)	3 (8)	1 (3)	31 (89)	3 (9)	1 (3)
* To what extent are you actively trying to reduce your child’s sugar intake?*^d^						
Intervention (base, *n*=40; 3MFU, *n*=29)	15 (38)	23 (58)	2 (5)	13 (45)	13 (45)	3 (10)
Control (base, *n*=38; 3MFU, *n*=35)	18 (47)	18 (47)	2 (5)	16 (46)	16 (46)	3 (9)
* Do you look at food labels when buying food?*^d^^‡^						
Intervention (base, *n*=40)	11 (28)	27 (68)	2 (5)	--	--	--
Control (base, *n*=39)	18 (46)	20 (51)	1 (3)	--	--	--
* Does nutritional information on food labels affect your shopping choices?*^d^^‡^						
Intervention (base, *n*=40)	7 (18)	28 (70)	5 (13)	--	--	--
Control (base, *n*=39)	14 (36)	25 (64)	0	--	--	--
*COM-B measures: Social opportunity*						
* How easy or difficult do you think your lifestyle makes it for you to limit your child’s sugar intake to the above guidelines, a day?*^e^						
Intervention (base, *n*=40; 3MFU, *n*=29)	15 (38)	10 (25)	15 (38)	9 (31)	5 (17)	15 (52)
Control (base, *n*=38; 3MFU, *n*=35)	20 (53)	11 (29)	7 (18)	19 (54)	10 (29)	6 (17)
* If you wanted advice or information on how to cut down on your child’s sugar consumption, do you know where to go?*^h^						
Intervention (3MFU, *n*=29)	--	--	--	20 (69)	4 (14)	5 (17)
Control (3MFU, *n*=35)	--	--	--	26 (74)	2 (6)	7 (20)

^a^Original response options and 3-point categorisation: Extremely important, very important [high agreeability], moderately important [medium agreeability], slightly important, not at all important [low agreeability]

^b^Original response options and 3-point categorisation: strongly agree, somewhat agree [high agreeability], neither agree nor disagree [medium agreeability], somewhat disagree, strongly disagree [low agreeability]

^c^Original response options and 3-point categorisation: Almost total control, a lot of control [high agreeability], moderate control [medium agreeability], a little bit of control, no control at all [low agreeability]

^d^Original response options and 3-point categorisation: Always, most of the time [high agreeability], about half the time [medium agreeability], sometimes, never [low agreeability]

^e^Original response options and 3-point categorisation: Extremely easy, somewhat easy [high agreeability], neither easy nor difficult [medium agreeability], somewhat difficult, extremely difficult [low agreeability]

^f^Original response options and 3-point categorisation: A great deal, a lot [high agreeability], a moderate amount [medium agreeability], a little, not at all [low agreeability]

^g^Original response options and 3-point categorisation: Extremely [high agreeability], very moderately, sightly [medium agreeability], not at all [low agreeability]

^h^Original response options and 3-point categorisation: Definitely yes, probably yes [high agreeability], might or might not [medium agreeability], probably not, definitely not [low agreeability]

^†^At 3-month follow up, the question was presented in the context of the Food Scanner app, for those in the intervention arm

^‡^Only measured at baseline

Regarding opportunities for behaviour change, in the intervention group, difficulty in limiting children's sugar intake increased from 38% at baseline to 52% at 3MFU. Automatic motivation to reduce sugar intake rose (50% to 62%), while reflective motivation declined (80% to 69%). No changes were observed in the control group. At baseline, 28% of the intervention group and 46% of the control group regularly checked food labels, with 18% vs. 36% reporting that labels influenced their shopping choices.

Attitudes remained stable between baseline and 3MFU in both groups. However, perceived control over children's sugar consumption declined in both conditions (intervention: 78% to 68%, control: 85% to 69%). Paired samples t-tests found no significant changes in psychological predictors over time in either group (see Additional File 3).

### Potential external influences

Most participants reported their child ate more snacks (61%), home-cooked meals (76%), and spent more on food (72%) during COVID-19. Additional findings on COVID-19 and other external influences, including physical activity, are in Additional File 4.

## Discussion

The current study set out to investigate the feasibility and acceptability of evaluating the Change4Life Food Scanner app in reducing children’s energy and sugar intake at 1-month and 3-month follow-up. The study additionally aimed to explore app engagement and changes in psychological outcomes over the study period. Despite the large drop-out rate, the study was considered feasible, acceptable, and sustainable amongst study completers. Preliminary analyses showed little evidence of improved psychological predictors of behaviour change within the intervention arm. Based on the pilot data, the app did not help reduce child energy (kcal) or sugar (g) intake via parental behaviour change.

Study feasibility was investigated through numerous methods. Participant recruitment and retention showed almost 30% attrition immediately after consenting procedures, and a further 20% drop out at 3MFU. In addition, a greater proportion of those randomised into the intervention condition dropped out before intervention exposure. Similar attrition rates have been reported within app-based interventions, calling for improved strategies to retain participants [53–55]. In addition, as the study was disrupted by COVID-19, the pandemic may have interfered with participant availability and willingness to commit to a 3-month trial and associated tasks. COVID-19 has previously been reported to disrupt study recruitment [56], data completeness and participant retention [57]. Further incentivisation approaches and behavioural insights may help counter participant attrition within a full-scale trial.

Study completers rated the study and intervention positively, but myfood24® had usability issues. Despite its extensive database [58], participants found it overwhelming and not smartphone-friendly at the time. These factors should be considered when choosing a suitable platform to log food diaries for a large-scale trial.

Preliminary analysis found no evidence that the Change4Life Food Scanner app reduced child energy or sugar intake compared to the control group. Both groups showed intake reductions, but these were larger (though non-significant) in the control arm. Given the small sample, inferential statistics were exploratory rather than conclusive, with effect sizes indicating little to no impact of the intervention. Evidence regarding the effectiveness of dietary digital interventions suggest potential modest effects [59–62]. Study findings add to a natural experiment that explored the effectiveness of the Change4Life campaign, which included a preceded version of the Food Scanner app. The campaign led to immediate, but not long-term reductions in children’s sugar consumption [20]. There was no control comparator to determine if reductions were due to the campaign. The results of this current study suggested reductions in food intake in both control and intervention arms, highlighting the importance of comparative research in this domain. Previous research additionally investigated BCTs adopted within the Food Scanner app [21]. The app used a variety of BCTs with evidence of effectiveness within obesity prevention and dietary interventions. However, it’s possible that the use and effectiveness of BCTs within lifestyle/behavioural interventions may not translate to app-based interventions.

There are many potential reasons for the lack of intervention effects. For instance, declining engagement with the Food Scanner app, both in usage time and items scanned. These findings are reflective of existing research on digital interventions [34, 63–65], where gradual engagement drop-off has been observed despite the inclusion of recommended app features [7]. Limited app exposure may have reduced participants’ interaction with key features and BCTs related to behavioural changes [66, 67]. As an independent evaluation, the study could not collect direct app usage data to accurately measure BCT exposure [68]. Understanding how and where the app was used would provide valuable context into how users interacted with the app. Incorporating these measures within a survey ought to be considered within a future trial [69]. Finally, variances between study conditions may have influenced dietary differences. For instance, there was a higher percentage of male children in the intervention arm, who have greater energy requirements [70]. Baseline differences in psychological predictors between conditions also emerged, despite random allocation. Control participants indicated greater nutritional awareness and tracking of child sugar consumption than those in the intervention condition. The self-monitoring nature of food diaries may have unintentionally promoted healthier eating behaviours within control participants [71]. Future studies could use a minimization method to better balance sociodemographics and other key variables between groups during the randomisation process [72].

COVID-19 and associated lockdown was an unforeseen event that occurred during the feasibility study. Study findings suggested that most children’s diets had been reportedly impacted by the pandemic, including parental food purchasing behaviours and greater snacking. Similar findings suggested that 48% of UK-based adults had increased food intake during the COVID-19 lockdown [49]. Research outside the UK has shown a significant increase in sugary drink consumption [73], as well as increased purchasing of ultra-processed cupboard staples [74]. This study’s preliminary findings may not be generalisable to a non-pandemic context or when conducting a full-scale trial.

App design and content may impact on app engagement thus effectiveness in improving dietary outcomes [75]. Most respondents liked the app and thought it was helpful. However, the app did not reportedly impact on the majority’s food shopping choices. This may have been due to several reasons. Firstly, the app did not recognise barcode scanned items from more affordable supermarkets. This is a fundamental limitation given that more people are now living in poverty and experiencing food insecurity due to the UK cost of living crisis [76, 77], and consequently relying on more affordable supermarkets than before [78]. Barcode scanner detection of foods within more affordable supermarkets may widen the app’s reach and increase engagement. In addition, respondents highlighted that the app was somewhat burdensome to use. During COVID-19, sanitisation of hands and inanimate objects became usual practices among the public to decrease virus transmission. Use of the Food Scanner app in store during COVID would have led to greater contact with unnecessary products and increased risk of infection. Therefore, people may have not used the app at the point of purchase.

There are several implications for app improvement and future research. Reformulation of front of pack nutritional labels may reduce the burden of using the Food Scanner app. This could include images of sugar cubes or teaspoons so that the public can benefit from easy-to-interpret nutritional information [79–81]. In addition, the incorporation of incentives such as access to money-off vouchers for healthier products was recommended. This would help offer a solution to the barriers (e.g. greater costs) of purchasing healthier alternatives [82].

### Strengths and limitations

The current study has provided rich quantitative and qualitative data on various aspects of the Change4Life Food Scanner app and the feasibility of the current evaluation approach. Study procedures and materials were informed by stakeholders [22, 29], and PPI, and were piloted through cognitive debriefing. Despite this, the feasibility study demonstrated high drop-out rates, resulting in several additional limitations. Acceptability and sustainability (long-term trial engagement) of study procedures are biased towards study completers. Since withdrawal feedback was obtained from a minority of participant dropouts, further insight into study acceptability amongst dropouts may be warranted. Study tasks were considerably long and potentially burdensome for participants, contributing to the high dropouts. Due to the small sample size, the study was not sufficiently powered to account for potential covariates within analyses, which may have led to different statistical outcomes. Small sample sizes also meant that it was not possible to generate additional comparisons of characteristics between users and non-users of the app, nor a comparison of app effects by sociodemographic groups. For instance, the majority of the sample were White and educated, and therefore the sample underrepresented groups most affected by obesity [83] who may have benefited most from using the Food Scanner app. Involving diverse populations in app development and research design through co-production may improve participant retention.

Outcome measures may have restricted observation of app engagement and intervention effects. Firstly, although parents were instructed to use the app, it is unknown whether children participated. Future evaluations would benefit by exploring child involvement in dietary app use in family-focused interventions. Secondly, intervention participants were not asked about changes in food purchases. Although the intervention was not superior to a control condition based on average data, changes may have been made to food purchases that could be impactful at a population level [84]. It would be beneficial for a full-scale trial to consider changes in food purchasing choices to capture all potential impacts of the Food Scanner app. Thirdly, despite 3-day food diary measures, food diary data was averaged regardless of how many food diaries were completed, as a method to preserve the sample size. Food diaries based on a single entry could risk the reliability of data due to a lack of representation of typical dietary behaviour offered via 3-day food diaries. Three-day food diaries have also been found to reduce errors in reporting in comparison to other reporting methods [85].

## Conclusions

The approach undertaken to evaluate the Change4Life Food Scanner app in reducing children’s energy and sugar intake was feasible. The analysis did not offer evidence of Food Scanner app effectiveness for improving children’s diets in comparison to a control condition at both 1-month and 3-month follow-up. However, the small sample size and COVID-19 disruptions cautions the overinterpretation of inferential statistics. Findings from this study, such as participant retention rates could help inform sample size estimates for a future trial, whilst study acceptability findings could inform the reconsideration of measurement tools. This study also provides useful participant, user-informed recommendations to improve the Food Scanner app, and the barriers within the system that could prevent its use. Findings of a full-scale trial could aid public health campaigns and policy teams to revise the app’s content and accessibility issues to help maximise user engagement, raise awareness around food and nutrition, and improve children’s diets.

## Supplementary Information

Additional file 1 (docx). Change4Life Food Scanner app evaluation: survey questions and response options. A table outlining all survey questions and response options, alongside references.

Additional file 2 (docx). Demographics of study dropouts. A table outlining the characteristics of participants who did not complete the study.

Additional file 3 (docx). A Within-Subjects Comparison (Mean ±SD) of Psychological Predictors of Behaviour Change Between Baseline and 3-Month Follow-up. A table outlining mean differences between baseline and follow-up survey responses relating to psychological predictors of behaviour change.

Additional file 4 (docx). Potential external influencers. An extension of the results section reporting baseline physical activity, awareness of external food policies and the impact of the coronavirus pandemic.

## Data Availability

The datasets used and/or analysed during the current study are available from the corresponding author on reasonable request.

## Abbreviations

1MFU: 1-Month follow-up
3MFU: 3-Month follow-up
BCT: Behaviour Change Technique
RCT: Randomised controlled trial

## References

[1] Beverley B, David C, Kerry S J, Polly P, Caireen R, Toni S, et al. National Diet and Nutrition Survey Rolling programme Years 9 to 11 (2016/2017 to 2018/2019)-a survey carried out on behalf of Public Health England and the Food Standards Agency. 2020.

[2] Public Health England. Children consume more than a year’s worth of sugar in 6 months. https://www.gov.uk/government/news/children-consume-more-than-a-years-worth-of-sugar-in-6-months (2018). Accessed 13 May 2025.

[3] NHS Digital. Statistics on Obesity, Physical Activity and Diet, England, 2020. https://digital.nhs.uk/data-and-information/publications/statistical/statistics-on-obesity-physical-activity-and-diet/england-2020/part-6-diet-copy (2020). Accessed 13 May 2025.

[4] Public Health England. Government recommendations for energy and nutrients for males and females aged 1 – 18 years and 19+ years. https://assets.publishing.service.gov.uk/media/5a749fece5274a44083b82d8/government_dietary_recommendations.pdf (2016). Accessed 13 May 2025.

[5] Janssen F, Bardoutsos A, Vidra N. Obesity prevalence in the long-term future in 18 European countries and in the USA. Obes Facts. 2020;13(5):514–27. 33075798 10.1159/000511023 PMC7670332

[6] Delisle Nyström C, Sandin S, Henriksson P, Henriksson H, Maddison R, Löf M. A 12-month follow-up of a mobile-based (mHealth) obesity prevention intervention in pre-school children: the MINISTOP randomized controlled trial. BMC Public Health. 2018;18(1):1–7. 10.1186/s12889-018-5569-4 PMC5968487 29793467

[7] Pearson N, Finch M, Sutherland R, Kingsland M, Wolfenden L, Wedesweiler T, et al. An mHealth Intervention to Reduce the Packing of Discretionary Foods in Children’s Lunch Boxes in Early Childhood Education and Care Services: Cluster Randomized Controlled Trial. JMIR. 2022;24(3): e27760. 35297768 10.2196/27760 PMC8972115

[8] Vázquez-Paz AM, Michel-Nava RM, Delgado-Pérez EE, Lares-Michel M, Espinosa-Curiel IE. Parents’ mHealth App for Promoting Healthy Eating Behaviors in Children: Feasibility, Acceptability, and Pilot Study. J Med Syst. 2022;46(11):70. 36109423 10.1007/s10916-022-01860-w

[9] Lin Y, Mâsse LC. A look at engagement profiles and behavior change: A profile analysis examining engagement with the Aim2Be lifestyle behavior modification app for teens and their families. Prev Med Rep. 2021;24: 101565. 34976631 10.1016/j.pmedr.2021.101565 PMC8683902

[10] Ni Mhurchu C, Eyles H, Jiang Y, Blakely T. Do nutrition labels influence healthier food choices? Analysis of label viewing behaviour and subsequent food purchases in a labelling intervention trial. Appetite. 2018;121:360–5. 29191745 10.1016/j.appet.2017.11.105

[11] West JH, Belvedere LM, Andreasen R, Frandsen C, Hall PC, Crookston BT. Controlling Your “App”etite: How Diet and Nutrition-Related Mobile Apps Lead to Behavior Change. JMIR Mhealth Uhealth. 2017;5(7): e95. 28694241 10.2196/mhealth.7410 PMC5525004

[12] Ni Mhurchu C, Volkova E, Jiang Y, Eyles H, Michie J, Neal B, et al. Effects of interpretive nutrition labels on consumer food purchases: the Starlight randomized controlled trial. Am J Clin Nutr. 2017;105(3):695–704. 28148503 10.3945/ajcn.116.144956

[13] Nyström CD, Sandin S, Henriksson P, Henriksson H, Trolle-Lagerros Y, Larsson C, et al. Mobile-based intervention intended to stop obesity in preschool-aged children: the MINISTOP randomized controlled trial. Am J Clin Nutr. 2017;105(6):1327–35. 28446496 10.3945/ajcn.116.150995

[14] Public Health England. Public Health England Social Marketing Strategy 2017 to 2020. https://assets.publishing.service.gov.uk/government/uploads/system/uploads/attachment_data/file/646715/public_health_england_marketing_strategy_2017_to_2020.pdf (2017). Accessed 13 May 2025.

[15] Croker H, Packer J, Russell SJ, Stansfield C, Viner R. Front of pack nutritional labelling schemes: a systematic review and meta-analysis of recent evidence relating to objectively measured consumption and purchasing. J Hum Nutr Diet. 2020;33(4):518–37. 32364292 10.1111/jhn.12758

[16] Machin L, Aschemann-Witzel J, Curutchet MR, Gimenez A, Ares G. Does front-of-pack nutrition information improve consumer ability to make healthful choices? Performance of warnings and the traffic light system in a simulated shopping experiment. Appetite. 2018;121:55–62. 29102533 10.1016/j.appet.2017.10.037

[17] Adams JM, Hart W, Gilmer L, Lloyd-Richardson EE, Burton KA. Concrete images of the sugar content in sugar-sweetened beverages reduces attraction to and selection of these beverages. Appetite. 2014;83:10–8. 25108238 10.1016/j.appet.2014.07.027

[18] Scapin T, Fernandes AC, Curioni CC, Pettigrew S, Neal B, Coyle DH, et al. Influence of sugar label formats on consumer understanding and amount of sugar in food choices: a systematic review and meta-analyses. Nutr Rev. 2021;79(7):788–801. 33313917 10.1093/nutrit/nuaa108

[19] Miller C, Wright K, Dono J, Pettigrew S, Wakefield M, Coveney J, et al. “You can’t just eat 16 teaspoons of sugar so why would you drink 16 teaspoons’ worth of sugar?”: a qualitative study of young adults’ reactions to sugary drink warning labels. BMC Public Health. 2022;22(1):1–12. 35733102 10.1186/s12889-022-13648-1 PMC9219237

[20] Bradley J, Gardner G, Rowland MK, Fay M, Mann K, Holmes R, et al. Impact of a health marketing campaign on sugars intake by children aged 5–11 years and parental views on reducing children’s consumption. BMC Public Health. 2020;20(1):1–11. 32223751 10.1186/s12889-020-8422-5 PMC7104521

[21] Mahdi S, Michalik-Denny EK, Buckland NJ. An Assessment of Behavior Change Techniques in Two Versions of a Dietary Mobile Application: The Change4Life Food Scanner. Front Public Health. 2022;10: 803152. 35284376 10.3389/fpubh.2022.803152 PMC8904754

[22] Mahdi S, Buckland NJ, Chilcott J. Economic and health impacts of the Change4Life Food Scanner app: Findings from a randomized pilot and feasibility study. Front Nutr. 2023;10:1125542. 37006945 10.3389/fnut.2023.1125542 PMC10061026

[23] Lancaster GA, Dodd S, Williamson PR. Design and analysis of pilot studies: recommendations for good practice. J Evaluation Clin Pract. 2004;10(2):307–12. 10.1111/j..2002.384.doc.x 15189396

[24] Thabane L, Ma J, Chu R, Cheng J, Ismaila A, Rios LP, et al. A tutorial on pilot studies: the what, why and how. BMC Med Res Methodol. 2010;10(1):1. 20053272 10.1186/1471-2288-10-1 PMC2824145

[25] Lancaster GA. Pilot and feasibility studies come of age! Pilot Feasibility Stud. 2015;1:1–4. 10.1186/2055-5784-1-1 PMC5842886 29611687

[26] Eldridge SM, Chan CL, Campbell MJ, Bond CM, Hopewell S, Thabane L, et al. CONSORT 2010 statement: extension to randomised pilot and feasibility trials. Pilot Feasibility Stud. 2016;2(1):64. 27965879 10.1186/s40814-016-0105-8 PMC5154046

[27] Viechtbauer W, Smits L, Kotz D, Budé L, Spigt M, Serroyen J, et al. A simple formula for the calculation of sample size in pilot studies. J Clin Epidemiol. 2015;68(11):1375–9. 26146089 10.1016/j.jclinepi.2015.04.014

[28] Robinson E, Kersbergen I, Brunstrom JM, Field M. I’m watching you. Awareness that food consumption is being monitored is a demand characteristic in eating-behaviour experiments. Appetite. 2014;83:19–25. 25086209 10.1016/j.appet.2014.07.029

[29] Mahdi S. An Investigation of Evaluation Approaches for Dietary Digital Interventions for Improving Children's Dietary Intake. [PhD thesis]: University of Sheffield; 2023.

[30] York Health Economics Consortium. Cognitive Debriefing. https://www.yhec.co.uk/glossary/cognitive-debriefing/ (2016). Accessed 13 May 2025.

[31] Department for Work and Pensions. Income distribution. https://www.ethnicity-facts-figures.service.gov.uk/work-pay-and-benefits/pay-and-income/income-distribution/latest#download-the-data (2022). Accessed 13 May 2025.

[32] Reale S, Kearney CM, Hetherington MM, Croden F, Cecil JE, Carstairs SA, et al. The feasibility and acceptability of two methods of snack portion control in United Kingdom (UK) preschool children: Reduction and replacement. Nutrients. 2018;10(10):1493. 30322090 10.3390/nu10101493 PMC6212871

[33] Chai LK, Collins CE, May C, Ashman A, Holder C, Brown LJ, et al. Feasibility and efficacy of a web-based family telehealth nutrition intervention to improve child weight status and dietary intake: a pilot randomised controlled trial. J Telemed Telecare. 2021;27(3):146–58. 31364474 10.1177/1357633X19865855

[34] Sutherland R, Nathan N, Brown A, Yoong S, Finch M, Lecathelinais C, et al. A randomized controlled trial to assess the potential efficacy, feasibility and acceptability of an m-health intervention targeting parents of school aged children to improve the nutritional quality of foods packed in the lunchbox “SWAP IT.” Int J Behav Nutr Phys Act. 2019;16(1):54. 31266506 10.1186/s12966-019-0812-7 PMC6604241

[35] Buckland NJ, Camidge D, Croden F, Myers A, Lavin JH, Stubbs RJ, et al. Women with a low-satiety phenotype show impaired appetite control and greater resistance to weight loss. Br J Nutr. 2019;122(8):951–9. 31340872 10.1017/S000711451900179X

[36] Neal B, Crino M, Dunford E, Gao A, Greenland R, Li N, et al. Effects of Different Types of Front-of-Pack Labelling Information on the Healthiness of Food Purchases-A Randomised Controlled Trial. Nutrients. 2017;9(12):1284. 10.3390/nu9121284 PMC5748735 29186803

[37] Villaseñor A, Cadmus-Bertram L, Patterson RE. Chapter 7 - Overview of Nutritional Epidemiology. In: Coulston AM, Boushey CJ, Ferruzzi MG, Delahanty LM, editors. Nutrition in the Prevention and Treatment of Disease (Fourth Edition): Elsevier; 2017. p. 145–65.

[38] Wark PA, Hardie LJ, Frost GS, Alwan NA, Carter M, Elliott P, et al. Validity of an online 24-h recall tool (myfood24) for dietary assessment in population studies: comparison with biomarkers and standard interviews. BMC Med. 2018;16(1):136. 30089491 10.1186/s12916-018-1113-8 PMC6083628

[39] Hutchesson MJ, Rollo ME, Callister R, Collins CE. Self-monitoring of dietary intake by young women: online food records completed on computer or smartphone are as accurate as paper-based food records but more acceptable. J Acad Nutr Diet. 2015;115(1):87–94. 25262244 10.1016/j.jand.2014.07.036

[40] Michie S, van Stralen MM, West R. The behaviour change wheel: a new method for characterising and designing behaviour change interventions. Implementation Sci. 2011;6:42. 10.1186/1748-5908-6-42 PMC3096582 21513547

[41] Ajzen I, Madden TJ. Prediction of goal-directed behavior: Attitudes, intentions, and perceived behavioral control. J Exp Soc Psychol. 1986;22(5):453–74.

[42] Stevens KJ. Working with children to develop dimensions for a preference-based, generic, pediatric, health-related quality-of-life measure. Qual Health Res. 2010;20(3):340–51. 20054040 10.1177/1049732309358328

[43] Ratcliffe J, Huynh E, Stevens K, Brazier J, Sawyer M, Flynn T. Nothing about us without us? A comparison of adolescent and adult health-state values for the child health utility-9D using profile case best–worst scaling. Health Econ. 2016;25(4):486–96. 25689621 10.1002/hec.3165

[44] Cottrell DJ, Wright-Hughes A, Collinson M, Boston P, Eisler I, Fortune S, et al. Effectiveness of systemic family therapy versus treatment as usual for young people after self-harm: a pragmatic, phase 3, multicentre, randomised controlled trial. Lancet Psychiatry. 2018;5(3):203–16. 29449180 10.1016/S2215-0366(18)30058-0 PMC5835764

[45] Powell CVE, Kolamunnage-Dona R, Lowe J, Boland A, Petrou S, Doull I, et al. MAGNEsium Trial In Children (MAGNETIC): a randomised, placebo-controlled trial and economic evaluation of nebulised magnesium sulphate in acute severe asthma in children. Health Technol Assess (Winchester, England). 2013;17(45):v. 10.3310/hta17450 PMC4781380 24144222

[46] Beecham J, Knapp M. Costing psychiatric interventions. In: Thornicroft, Graham, ed. Measuring Mental Health Needs (Second Edition). Royal College of Psychiatrists, London: Cambridge University Press; 2001. p. 200–24.

[47] Carroll JK, Moorhead A, Bond R, LeBlanc WG, Petrella RJ, Fiscella K. Who Uses Mobile Phone Health Apps and Does Use Matter? A Secondary Data Analytics Approach. J Med Internet Res. 2017;19(4): e125. 28428170 10.2196/jmir.5604 PMC5415654

[48] NHS. Physical activity guidelines for children and young people. https://www.nhs.uk/live-well/exercise/physical-activity-guidelines-children-and-young-people/ (2021). Accessed 13 May 2025.

[49] Buckland NJ, Swinnerton LF, Ng K, Price M, Wilkinson LL, Myers A, et al. Susceptibility to increased high energy dense sweet and savoury food intake in response to the COVID-19 lockdown: The role of craving control and acceptance coping strategies. Appetite. 2021;158: 105017. 33161044 10.1016/j.appet.2020.105017 PMC8580210

[50] Braun V, Clarke V. Using thematic analysis in psychology. Qual Res Psychol. 2006;3(2):77–101.

[51] Howell DC, Rogier M, Yzerbyt V, Bestgen Y. Statistical methods in human sciences. New York: Wadsworth; 1998. p. 721.

[52] Shanyinde M, Pickering RM, Weatherall M. Questions asked and answered in pilot and feasibility randomized controlled trials. BMC Med Res Methodol. 2011;11(1):117. 21846349 10.1186/1471-2288-11-117 PMC3170294

[53] Meyerowitz-Katz G, Ravi S, Arnolda L, Feng X, Maberly G, Astell-Burt T. Rates of attrition and dropout in app-based interventions for chronic disease: systematic review and meta-analysis. J Med Internet Res. 2020;22(9): e20283. 32990635 10.2196/20283 PMC7556375

[54] Sousa P, Martinho R, Reis CI, Dias SS, Gaspar PJ, Dixe MdA, et al. Controlled trial of an mHealth intervention to promote healthy behaviours in adolescence (TeenPower): Effectiveness analysis. J Adv Nurs. 2020;76(4):1057–68. 10.1111/jan.14301 31880009

[55] Jakob R, Harperink S, Rudolf AM, Fleisch E, Haug S, Mair JL, et al. Factors influencing adherence to mHealth apps for prevention or management of noncommunicable diseases: systematic review. J Med Internet Res. 2022;24(5): e35371. 35612886 10.2196/35371 PMC9178451

[56] Sathian B, Asim M, Banerjee I, Pizarro AB, Roy B, Van Teijlingen ER, et al. Impact of COVID-19 on clinical trials and clinical research: a systematic review. Nepal J Epidemiol. 2020;10(3):878. 33042591 10.3126/nje.v10i3.31622 PMC7538012

[57] Jose K, Sharman M, Stanesby O, Greaves S, Venn A, Blizzard L, et al. Incentivising public transport use for physical activity gain: process evaluation of the COVID-19 disrupted trips4health randomised controlled trial. Int J Behav Nutr Phys Act. 2022;19(1):1–13. 36550500 10.1186/s12966-022-01394-x PMC9772596

[58] myfood24. https://www.myfood24.org/ (2024). Accessed 13 May 2025.

[59] Langarizadeh M, Sadeghi M, As’habi A, Rahmati P, Sheikhtaheri A. Mobile apps for weight management in children and adolescents; An updated systematic review. Patient Educ Couns. 2021;104(9):2181–8. 10.1016/j.pec.2021.01.035 33573915

[60] Yau KW, Tang TS, Görges M, Pinkney S, Kim AD, Kalia A, et al. Effectiveness of Mobile Apps in Promoting Healthy Behavior Changes and Preventing Obesity in Children: Systematic Review. JMIR Pediatr Parent. 2022;5(1): e34967. 35343908 10.2196/34967 PMC9002598

[61] Bonvicini L, Pingani I, Venturelli F, Patrignani N, Bassi MC, Broccoli S, et al. Effectiveness of mobile health interventions targeting parents to prevent and treat childhood Obesity: Systematic review. Prev Med Rep. 2022;29:101940. 10.1016/j.pmedr.2022.101940 PMC9501985 36161123

[62] Islam MM, Poly TN, Walther BA, Li Y-C. Use of mobile phone app interventions to promote weight loss: meta-analysis. JMIR Mhealth Uhealth. 2020;8(7): e17039. 32706724 10.2196/17039 PMC7407260

[63] Schoeppe S, Alley S, Van Lippevelde W, Bray NA, Williams SL, Duncan MJ, et al. Efficacy of interventions that use apps to improve diet, physical activity and sedentary behaviour: a systematic review. Int J Behav Nutr Phys Act. 2016;13(1):127. 27927218 10.1186/s12966-016-0454-y PMC5142356

[64] Vaghefi I, Tulu B. The continued use of mobile health apps: insights from a longitudinal study. JMIR Mhealth Uhealth. 2019;7(8): e12983. 31469081 10.2196/12983 PMC6740166

[65] Russell CG, Denney-Wilson E, Laws RA, Abbott G, Zheng M, Lymer SJ, et al. Impact of the growing healthy mHealth program on maternal feeding practices, infant food preferences, and satiety responsiveness: quasi-experimental study. JMIR Mhealth Uhealth. 2018;6(4): e9303. 10.2196/mhealth.9303 PMC5943630 29695373

[66] Gilliland J, Sadler R, Clark A, O’Connor C, Milczarek M, Doherty S. Using a Smartphone Application to Promote Healthy Dietary Behaviours and Local Food Consumption. BioMed Res Int. 2015;2015: 841368. 26380298 10.1155/2015/841368 PMC4561980

[67] Villinger K, Wahl DR, Boeing H, Schupp HT, Renner B. The effectiveness of app-based mobile interventions on nutrition behaviours and nutrition-related health outcomes: A systematic review and meta-analysis. Obes Rev. 2019;20(10):1465–84. 31353783 10.1111/obr.12903 PMC6852183

[68] Murray E, Hekler EB, Andersson G, Collins LM, Doherty A, Hollis C, et al. Evaluating Digital Health Interventions: Key Questions and Approaches. Am J Prev Med. 2016;51(5):843–51. 27745684 10.1016/j.amepre.2016.06.008 PMC5324832

[69] Perski O, Blandford A, Garnett C, Crane D, West R, Michie S. A self-report measure of engagement with digital behavior change interventions (DBCIs): development and psychometric evaluation of the “DBCI Engagement Scale.” Transl Behav Med. 2020;10(1):267–77. 30927357 10.1093/tbm/ibz039 PMC8411853

[70] NHS. How many calories does a child of 7 to 10 need? https://www.nhs.uk/common-health-questions/childrens-health/how-many-calories-does-a-child-of-7-10-need/ (2021). Accessed 13 May 2025.

[71] Michie S, Abraham C, Whittington C, McAteer J, Gupta S. Effective techniques in healthy eating and physical activity interventions: a meta-regression. Health Psychol. 2009;28(6):690. 19916637 10.1037/a0016136

[72] Altman DG, Bland JM. Treatment allocation by minimisation. BMJ. 2005;330(7495):843. 15817555 10.1136/bmj.330.7495.843 PMC556084

[73] Pietrobelli A, Pecoraro L, Ferruzzi A, Heo M, Faith M, Zoller T, et al. Effects of COVID-19 lockdown on lifestyle behaviors in children with obesity living in Verona, Italy: a longitudinal study. Obes. 2020;28(8):1382–5. 10.1002/oby.22861 PMC7267384 32352652

[74] Skerritt J, Mulvany L, Almeida I. Americans drop kale and quinoa to lock down with chips and oreos. Bloomberg News. https://www.bloomberg.com/news/articles/2020-03-21/americans-drop-kale-and-quinoa-to-lock-down-with-chips-and-oreos (2020). Accessed 13 May 2025.

[75] Perski O, Blandford A, West R, Michie S. Conceptualising engagement with digital behaviour change interventions: a systematic review using principles from critical interpretive synthesis. Transl Behav Med. 2017;7(2):254–67. 27966189 10.1007/s13142-016-0453-1 PMC5526809

[76] The Food Foundation. Millions of adults missing meals as cost of living crisis bites. https://foodfoundation.org.uk/press-release/millions-adults-missing-meals-cost-living-crisis-bites (2022). Accessed 13 May 2025.

[77] Bisdounis L. Cost of living: The healthcare ecosystem. UK Parliament. https://lordslibrary.parliament.uk/cost-of-living-the-healthcare-ecosystem/#:~:text=The%20rising%20cost%20of%20living%20has%20also%20impacted%20food%20security,banks%20reported%20a%20demand%20rise (2022). Accessed 13 May 2025.

[78] Farooqui JB. Aldi sales grow 19% as cost-of-living crisis sees surge in customers. Proactive. https://www.proactiveinvestors.co.uk/companies/news/993633/aldi-sales-grow-19-as-cost-of-living-crisis-sees-surge-in-customers-993633.html (2022). Accessed 13 May 2025.

[79] Lilo EA, West A. “OMG, I Get Like 100 Teaspoons of Sugar a Day!” Rural Teens’ Grasp of Their Beverage Consumption Habits. Community Health Equity Res Policy. 2022;42(4):367–73. 10.1177/0272684X211004928 33752544

[80] Bleich SN, Barry CL, Gary-Webb TL, Herring BJ. Reducing sugar-sweetened beverage consumption by providing caloric information: how black adolescents alter their purchases and whether the effects persist. Am J Public Health. 2014;104(12):2417–24. 25322298 10.2105/AJPH.2014.302150 PMC4232169

[81] Billich N, Blake MR, Backholer K, Cobcroft M, Li V, Peeters A. The effect of sugar-sweetened beverage front-of-pack labels on drink selection, health knowledge and awareness: An online randomised controlled trial. Appetite. 2018;128:233–41. 29879450 10.1016/j.appet.2018.05.149

[82] Goudie S, Hughes I. The Broken Plate 2022 The State of the Nation's Food System. The Food Foundation. https://foodfoundation.org.uk/sites/default/files/2023-01/FF_Broken_Plate_Report%202022_DIGITAL_UPDATED_2023.pdf (2022). Accessed 13 May 2025.

[83] NHS Digital. National Child Measurement Programme, England, 2021/22 school year. https://digital.nhs.uk/data-and-information/publications/statistical/national-child-measurement-programme/2021-22-school-year (2022). Accessed 13 May 2025.

[84] Cleghorn C, Wilson N, Nair N, Kvizhinadze G, Nghiem N, McLeod M, et al. Health Benefits and Cost-Effectiveness From Promoting Smartphone Apps for Weight Loss: Multistate Life Table Modeling. JMIR Mhealth Uhealth. 2019;7(1): e11118. 30664471 10.2196/11118 PMC6350086

[85] Crawford PB, Obarzanek E, Morrison J, Sabry Z. Comparative advantage of 3-day food records over 24-hour recall and 5-day food frequency validated by observation of 9-and 10-year-old girls. J Am Diet Assoc. 1994;94(6):626–30. 8195550 10.1016/0002-8223(94)90158-9

