# Supplementary material for: The impact of the Change4Life Food Scanner app on children’s diets and parental psychological outcomes: a randomised pilot and feasibility study
Source: BMC Public Health. 2025 Jul 2;25:2215. doi: 10.1186/s12889-025-23400-0 (PMC12220540; doi:10.1186/s12889-025-23400-0)
Supplement: Supplementary file 1 — Additional file 1 (docx). Change4Life Food Scanner app evaluation: survey questions and response options. A table outlining all survey questions and response options, alongside references. [file 12889_2025_23400_MOESM1_ESM.docx]

**Additional File 1 – Change4Life Food Scanner app evaluation: survey questions and response options**

| **Question** | **Answers** |
| --- | --- |
| **BASELINE *– ALL PARTICIPANTS*** |  |
|  |  |
| **Psychological predictors: Attitudes*** |  |
| How important is it for you that your family eat a healthy diet? ‡ | Extremely important/ Very important/ Moderately important/ Slightly important/ Not at all important |
|  |  |
| Please rate how much you agree with the following statements:   1. Having too much sugar leads to disease (West et al., 2017) 2. When buying food, snacks or drinks for my child, it is important to pay attention to the amount of sugar it contains (Chien et al., 2018) 3. For my child to be healthy, I need to be careful how much *saturated fat* my child eats‡ 4. For my child to be healthy, I need to be careful how much *sugar* my child eats‡ 5. For my child to be healthy, I need to be careful how many *calories* my child eats‡ | Strongly agree/ Somewhat agree/ Neither agree nor disagree/ Somewhat disagree/ Strongly disagree |
|  |  |
|  |  |
| **Psychological predictors: Perceived Behavioural Control*** |  |
| How much control do you have over your child’s sugar consumption? (Povey et al., 2000) | Almost total control/ A lot of control/ Moderate control/ A little bit of control/ No control at all |
|  |  |
| **COM-B MODEL** |  |
| **Psychological predictors: Physical capability** |  |
| The Government's recommended daily guidelines for child sugar intake is:  4-6 years: 19 grams  7-10 years: 24 grams  11+ years: 30 grams  *for reference, a standard 330ml can of coca cola contains 35g sugar |  |
| How often, if at all, do you keep track of how much sugar your child eats or drinks each day?* (Stevely et al., 2018) | Always/ Most of the time/ About half the time/ Sometimes/ Never |
|  |  |
| **Psychological predictors: Psychological capability** |  |
| “Too much sugar intake for my child increases their risk of obesity”* (Chien et al., 2018) | Strongly agree/ Somewhat agree/ Neither agree nor disagree/ Somewhat disagree/ Strongly disagree |
|  |  |
| “Nutritional labels are hard to understand” † ((Méjean et al., 2013) | Strongly agree/ Somewhat agree/ Neither agree nor disagree/ Somewhat disagree/ Strongly disagree/ I did not know there was a nutritional food label |
|  |  |
| How easy or difficult do you find it to limit your child's sugar intake to the amounts recommended in the above guidelines?* (Stevely et al., 2018) | Extremely easy/ Somewhat easy/ Neither easy nor difficult/ Somewhat difficult/ Extremely difficult/ I don’t know how much sugar my child consumes |
|  |  |
| How much do you think you know about making healthy food choices? †‡ | A great deal/ A lot/ A moderate amount/ A little/ None at all |
|  |  |
| **Psychological predictors: Capability (knowledge)** |  |
| What do you think is the daily-recommended sugar intake for your child's age, in grams?* (Stevely et al., 2018) | Open ended response  Not sure |
|  |  |
|  |  |
| **Psychological predictors: Social opportunity** (Stevely et al., 2018) |  |
| How easy or difficult do you think your lifestyle makes it for you to limit your child's sugar intake to the above guidelines, a day?* | Extremely easy/ Somewhat easy/ Neither easy nor difficult/ Somewhat difficult/ Extremely difficult |
|  |  |
| **Psychological predictors: Automatic motivation** (Stevely et al., 2018) |  |
| How concerned, if at all, are you about your child consuming more sugar than what is recommended?* | Extremely/ Very/ Moderately/ Slightly/ Not at all |
|  |  |
| To what extent do you want to keep your child's sugar consumption within recommended guidelines?* | Extremely/ Very/ Moderately/ Slightly/ Not at all |
|  |  |
| **Psychological predictors: Reflective motivation** |  |
| To what extent do you intend to keep your child's sugar consumption within recommended guidelines?* (Stevely et al., 2018) | Definitely yes/ Probably yes/ Might or might not/ Probably not/ Definitely not |
|  |  |
| To what extent are you actively trying to reduce your child's sugar intake?* (Stevely et al., 2018) | Always/ Most of the time/ Sometimes/ Rarely/ Never |
|  |  |
| *Food labels, also called nutrition labels, show how much sugar, saturated fat and salt are inside what we are buying. Food labels can be found on most food and drink, usually on the front of the pack.* |  |
| Do you look at food labels when buying food? ‡ | Always/ Most of the time/ About half the time/ Sometimes/ Never/ I did not know there was a nutritional food label |
|  |  |
| Does nutritional information on food labels affect your shopping choices? ((Kakinami et al., 2016) | Always/ Most of the time/ About half the time/ Sometimes/ Never |
| **Child Health Utility 9D instrument*** (Stevens, 2012) |  |
| These questions ask about how your child is today. For each question, read all the choices and decide which one is most like your child today. Only tick one box for each question. |  |
| Worried | My child doesn’t feel worried today/ My child feels a little bit worried today/ My child feels a bit worried today My child feels quite worried today/ My child feels very worried today |
|  |  |
| Sad | My child doesn’t feel sad today/ My child feels a little bit sad today/ My child feels a bit sad today/ My child feels quite sad today/ My child feels very sad today |
|  |  |
| Pain | My child doesn’t have any pain today/ My child has a little bit of pain today/ My child has a bit of pain today/ My child has quite a lot of pain today/ My child has a lot of pain today |
|  |  |
| Tired | My child doesn’t feel tired today/ My child feels a little bit tired today/ My child feels a bit tired today/ My child feels quite tired today/ My child feels very tired today |
|  |  |
| Annoyed | My child doesn’t feel annoyed today/ My child feels a little bit annoyed today/ My child feels a bit annoyed today/ My child feels quite annoyed today/ My child feels very annoyed today |
|  |  |
| School work/homework (such as reading, writing, doing lessons) | My child has no problems with their schoolwork/homework today/ My child has a few problems with their schoolwork/homework today/ My child has some problems with their schoolwork/homework today/ My child has many problems with their schoolwork/homework today/ My child can’t do the schoolwork/homework today |
|  |  |
| Sleep | Last night my child had no problems sleeping/ Last night my child had a few problems sleeping/ Last night my child had some problems sleeping/ Last night my child had many problems sleeping/ Last night my child couldn’t sleep at all |
|  |  |
| Daily routine (things like eating, having a bath/shower, getting dressed) | My child has no problems with their daily routine today/ My child has a few problems with their daily routine today/ My child has some problems with their daily routine today/ My child has many problems with their daily routine today/ My child can’t do their daily routine today |
|  |  |
| Able to join in activities (things like playing out with their friends, doing sports, joining in things) | My child can join in with any activities today/ My child can join in with most activities today/ My child can join in with some activities today/ My child can join in with a few activities today/ My child can join in with no activities  today |
| **Healthcare service use*** (Cottrell et al., 2018) |  |
| Please complete the following questions about your child's health.  Has your child used any of the following services in the last 3 months? |  |
| GP (family doctor)  Practice or district nurse  Hospital inpatient stay (staying in hospital overnight)  Hospital outpatient clinic(doctor visits, scans, other health professional)  Hospital accident and emergency department  Non-routine dentist or dental care | Yes/No  Yes/No  Yes/No  Yes/No  Yes/No  Yes/No |
|  |  |
| Questions repeated for each of the services above:  What are the total number of times your child used this service? | Open-ended question |
| What was the total length of time spent per contact (minutes)  Were you with your child during the visit? | Open-ended question  Yes/No |
|  |  |
| **School absenteeism/Workplace productivity*** (Powell et al., 2013, Beecham and Knapp, 2001) | |
| How many full days (or half days) has your child been absent from school because of health problems (e.g. attending hospital or seeing the family doctor) in the last 3 months? | Open-ended question |
| How many days have you been absent from work in the last 3 months? | Response options: 0-93 |
| Of these, how many are due to your child’s health? | Response options: 0-93 |
|  |  |
| **Physical activity** (Carroll et al., 2017) |  |
| Moderate intensity physical activity causes people to get warmer, breathe harder and their hearts to beat faster. |  |
| In a typical week how many days does your child do any physical activity or exercise of at least moderate intensity, such as brisk walking, bicycling at a regular pace, and swimming at a regular pace? | Daily/ 4-6 times a week/ 2-3 times a week/ Once a week/ Never |
| On the week days that your child does any physical activity or exercise of at least moderate intensity how long do they do these activities? | _____ hours/ _____minutes |
| On the weekend days that your child does any physical activity or exercise of at least moderate intensity how long do they do these activities? | _____ hours/ _____minutes |
|  |  |
| **Previous dietary app use**‡ |  |
| Please indicate which of the following apps you have previously used. | MyFitnessPal  Nootric  Change4Life Food Scanner  Lifesum  Change4Life Smart Recipes  FoodSwitch UK  Change4Life Sugar Smart  Other, please specify:  None |
| **FORTNIGHTLY APP ENGAGEMENT – *APP USERS ONLY*** ‡ |  |
|  |  |
| On how many days in the last 2 weeks did you use the app to help make food choices for your child? | ______ days (choices from 0-14) |
| On the days that you used the app, on average how much time (in minutes) did you spend using it? | _______ minutes |
| When using the Food Scanner app, how many items did you scan in the last 2 weeks? You can find a list of the last 20 items scanned through the app. | _______ |
|  |  |
| **3-MONTH FOLLOW UP – *ALL PARTICIPANTS (in addition to all questions at baseline marked with *)*** | |
|  |  |
| **Psychological predictors: COM-B model – Social opportunity** |  |
| If you wanted advice or information on how to cut down on your child’s sugar consumption, do you know where to go? (Stevely et al., 2018) | Definitely yes/ Probably yes/ Might or might not/ Probably not/ Definitely not |
|  |  |
| **COVID-19 and impact on diet** |  |
| To what extent do you feel that the lifestyle changes imposed by the Government in relation to the Coronavirus has affected the following:  Your child’s diet  Your ability to make healthier food choices for your child  Your food purchasing behaviour  The types of food you bought  Your participation in this study | A great deal/ A lot/ A moderate amount/ A little/ Not at all |
| “The lifestyle changes imposed by the Government in relation to the Coronavirus led my child to…” | Strongly agree/ Somewhat agree/ Neither agree nor disagree/ Somewhat disagree/ Strongly disagree |
| … eat more sugar than they did before |  |
| … eat more snacks than they did before  … eat more fruit and vegetables than they did before |  |
| … eat more home cooked meals than they did before |  |
| … be more physically active than they were before |  |
|  |  |
| To what extent do you feel that the lifestyle changes imposed by the Government in relation to the Coronavirus (COVID-19) has affected the following, in comparison to before the lockdown: | A lot less/ Slightly less/ The same/ Slightly more/ A lot more |
| Since the COVID-19 lockdown, I carry out online grocery shopping… |  |
| Since the COVID-19 lockdown, my children eat take out food… |  |
| Since the COVID-19 lockdown, I have been purchasing sugary foods or treats/snacks… |  |
| Since the COVID-19 lockdown, I have been spending on food… |  |
|  |  |
| Has the Coronavirus outbreak, or any other events, affected your responses or engagement in the trial? If yes, please detail. | Yes/No |
|  |  |
| **External policy confounders**‡ |  |
| Has the introduction of the sugar tax led you to buy different drinks for the household? | Always/ Most of the time/ About half the time/ Sometimes/ Never/ Do not know |
|  |  |
| Has the introduction of the sugar tax reduced your child’s sugar intake? | Always/ Most of the time/ About half the time/ Sometimes/ Never/ Do not know |
|  |  |
| Please indicate how much you agree with this statement:  “Existing public health campaigns and messages have helped me improve my child’s diet” | Strongly agree/ Somewhat agree/ Neither agree nor disagree/ Somewhat disagree/ Strongly disagree/ I am not aware of any public health campaigns or messages |
|  |  |
| How familiar are you with Change4Life? | Extremely familiar/ Very familiar/ Moderately familiar/ Slightly familiar/ Not familiar at all |
|  |  |
| Do you currently use Change4Life resources? | Always/ Most of the time/ About half the time/ Sometimes/ Never |
|  |  |
| Are there any other factors that may have had an influence over your child’s sugar consumption in the last 3 months? If yes, please specify. | Yes, please specify….  No |
| **Study acceptability and feasibility** (Reale et al., 2018) |  |
| To what extent was this study easy to complete? | Extremely easy/ Somewhat easy/ Neither easy nor difficult/ Somewhat difficult/ Extremely difficult |
|  |  |
| To what extent was participating in this study time consuming/demanding? | A great deal/ A lot/ A moderate amount/ A little/ None at all |
|  |  |
| Did you find that receiving reminders to complete food diaries and surveys helpful? | Strongly agree/ Somewhat agree/ Neither agree nor disagree/ Somewhat disagree/ Strongly disagree |
| Were you able to complete all requested study tasks? | Completed all the tasks/ Completed the majority of the tasks/ Completed a fair amount of the tasks/ Completed very few of the tasks |
|  |  |
| What prevented you from completing all study tasks? | Open-ended response |
|  |  |
| Was there anything we could have done to keep you more engaged in completing food diaries and surveys throughout this study? Please explain. | Open-ended response |
|  |  |
| **Food diary acceptability** (Buckland et al., 2019) |  |
| How has the food diary affected your child’s eating or what you have recorded generally over the past 3 months? | Strongly agree/ Somewhat agree/ Neither agree nor disagree/ Somewhat disagree/ Strongly disagree |
| I did not report everything my child ate |  |
| I changed what my child actually ate to make it easier to record |  |
| It had no effect on what my child ate |  |
| It was easy to use |  |
| I found it too much work |  |
|  |  |
| **Sustainability** |  |
| If this study was extended to a 12-month follow-up, would you be willing to continue for 9 more months? | Definitely yes/ Probably yes/ Might or might not/ Probably not/ Definitely not |
|  |  |
| Do you have any other comments you would like to make about the study? | Open-ended response |
| **3-MONTH FOLLOW UP – *APP USERS ONLY*** |  |
|  |  |
| **Psychological predictors: COM-B model – Physical capability** |  |
| Think about the nutrition app that you have used in the past 3 months.  “Using the app has increased my ability to reduce the number of high sugar snacks that my child eats” (West et al., 2017) | Strongly agree/ Somewhat agree/ Neither agree nor disagree/ Somewhat disagree/ Strongly disagree |
| “The Food Scanner app has helped me make healthier food choices for my child” ‡ | Strongly agree/ Somewhat agree/ Neither agree nor disagree/ Somewhat disagree/ Strongly disagree |
|  |  |
|  |  |
| **Psychological predictors: COM-B model – Psychological capability** |  |
| How much do you think you know about making healthy food choices after using the Food Scanner app? (Méjean et al., 2013) | A great deal/ A lot/ A moderate amount/ A little/ None at all |
| With the Food Scanner App, I find nutritional labels hard to understand (Méjean et al., 2013) | Strongly agree/ Somewhat agree/ Neither agree nor disagree/ Somewhat disagree/ Strongly disagree |
|  |  |
| **App engagement**‡ (Méjean et al., 2013) |  |
| Have you noticed any changes or updates in the Food Scanner app, in the past 3 months? | Yes/No |
| Has the latest Food Scanner app update improved your engagement with the app? | A great deal, a lot, a moderate amount, a little, none at all |
| **App likeability** (West et al., 2017) |  |
| The app was helpful | Strongly agree/ Somewhat agree/ Neither agree nor disagree/ Somewhat disagree/ Strongly disagree |
| The app was easy to use |  |
| I enjoyed using the app |  |
| I liked the app |  |
| I would recommend the app to others |  |
|  |  |
| **App usefulness** (Neal et al., 2017) |  |
| Did you use the Food Scanner app at least once throughout this study? | Yes/No |
|  |  |
| How useful did you find the sugar cube images shown in the app? | Extremely useful/ Very useful/ Moderately useful/ Slightly useful/ Not at all useful |
|  |  |
| How easy to understand were the sugar cube images shown in the app? | Extremely easy/ Somewhat easy/ Neither easy nor difficult/ Somewhat difficult/ Extremely difficult |
|  |  |
| How useful would it be to have those sugar cube images printed on food packages, as part of the nutritional label? | Extremely useful/ Very useful/ Moderately useful/ Slightly useful/ Not at all useful |
|  |  |
| How often did the Food Scanner app help you choose to buy different foods or drinks? | Always/ Most of the time/ About half the time/ Sometimes/ Never |
|  |  |
| **App consequences**‡ |  |
| Using the food scanner app has led me to spend _____ on groceries | A lot more/ Slightly more/ The same/ Slightly less/ A lot less |
|  |  |
| **App feedback – open ended questions** (Lieffers et al., 2018) |  |
| What did you like about the app? |  |
| What did you dislike about the app? |  |
| How can the app be improved to make it more attractive to use (e.g. app features)? |  |
| How can the app be improved to help you use it more often? |  |
| How can the app be improved to help support healthier eating behaviours? |  |
| Did anything prevent you from using the app? Please detail. |  |
|  |  |
| **COVID-19 and impact on diet**‡ |  |
| *To what extent do you feel that the lifestyle changes imposed by the Government in relating to the Coronavirus has affected the following:* | A great deal/ A lot/ A moderate amount/ A little/ Not at all |
| Your ability to scan barcodes using the Food Scanner app |  |
| Did the Food Scanner app support you at this time in making healthier food choices? |  |
| *Questions asked at both baseline and follow-up  † Questions asked at both baseline and follow-up for controls only  ‡ Question produced by the researcher for the purposes of this study |  |

**References**

1. Beecham, J. and Knapp, M. (2001) 'Costing psychiatric interventions', in Thornicroft, G. (ed.) *Measuring Mental Health Needs (Second Edition)*. Royal College of Psychiatrists, London, pp. 200-224.
2. Buckland, N. J., Camidge, D., Croden, F., Myers, A., Lavin, J. H., Stubbs, R. J., Blundell, J. E. and Finlayson, G. (2019) 'Women with a low-satiety phenotype show impaired appetite control and greater resistance to weight loss', *British Journal of Nutrition,* 122(8), pp. 951-959. DOI: <https://doi.org/10.1017/S000711451900179X>.
3. Carroll, J. K., Moorhead, A., Bond, R., LeBlanc, W. G., Petrella, R. J. and Fiscella, K. (2017) 'Who Uses Mobile Phone Health Apps and Does Use Matter? A Secondary Data Analytics Approach', *Journal of Medical Internet Research,* 19(4), pp. e125. DOI: <https://doi.org/10.2196/jmir.5604>.
4. Chien, T.-Y., Chien, Y.-W., Chang, J.-S. and Chen, Y. (2018) 'Influence of mothers’ nutrition knowledge and attitudes on their purchase intention for infant cereal with no added sugar claim', *Nutrients,* 10(4), pp. 435. DOI: <https://doi.org/10.3390/nu10040435>.
5. Cottrell, D. J., Wright-Hughes, A., Collinson, M., Boston, P., Eisler, I., Fortune, S., Graham, E. H., Green, J., House, A. O. and Kerfoot, M. (2018) 'Effectiveness of systemic family therapy versus treatment as usual for young people after self-harm: a pragmatic, phase 3, multicentre, randomised controlled trial', *The Lancet Psychiatry,* 5(3), pp. 203-216. DOI: <https://doi.org/10.1016/S2215-0366(18)30058-0>.
6. Kakinami, L., Houle-Johnson, S. and McGrath, J. J. (2016) 'Parental Nutrition Knowledge Rather Than Nutrition Label Use Is Associated With Adiposity in Children', *Journal of Nutrition Education and Behavior,* 48(7), pp. 461-467. DOI: <https://doi.org/10.1016/j.jneb.2016.04.005>.
7. Lieffers, J. R. L., Arocha, J. F., Grindrod, K. and Hanning, R. M. (2018) 'Experiences and Perceptions of Adults Accessing Publicly Available Nutrition Behavior-Change Mobile Apps for Weight Management', *Journal of the Academy of Nutrition Dietetics,* 118(2), pp. 229-239. DOI: <https://doi.org/10.1016/j.jand.2017.04.015>.
8. Méjean, C., Macouillard, P., Péneau, S., Hercberg, S. and Castetbon, K. (2013) 'Perception of front-of-pack labels according to social characteristics, nutritional knowledge and food purchasing habits', *Public Health Nutrition,* 16(3), pp. 392-402. DOI: <https://doi.org/10.1017/S1368980012003515>.
9. Neal, B., Crino, M., Dunford, E., Gao, A., Greenland, R., Li, N., Ngai, J., Ni Mhurchu, C., Pettigrew, S., Sacks, G., Webster, J. and Wu, J. H. (2017) 'Effects of Different Types of Front-of-Pack Labelling Information on the Healthiness of Food Purchases-A Randomised Controlled Trial', *Nutrients,* 9(12). DOI: <https://doi.org/10.3390/nu9121284>.
10. Povey, R., Conner, M., Sparks, P., James, R. and Shepherd, R. (2000) 'Application of the Theory of Planned Behaviour to two dietary behaviours: Roles of perceived control and self‐efficacy', *British Journal of Health Psychology,* 5(2), pp. 121-139. DOI: <https://doi.org/10.1348/135910700168810>.
11. Powell, C. V. E., Kolamunnage-Dona, R., Lowe, J., Boland, A., Petrou, S., Doull, I., Hood, K., Williamson, P. and group, M. s. (2013) 'MAGNEsium Trial In Children (MAGNETIC): a randomised, placebo-controlled trial and economic evaluation of nebulised magnesium sulphate in acute severe asthma in children', *Health Technology Assessment (Winchester, England),* 17(45), pp. v. DOI: <https://doi.org/10.3310/hta17450>.
12. Reale, S., Kearney, C. M., Hetherington, M. M., Croden, F., Cecil, J. E., Carstairs, S. A., Rolls, B. J. and Caton, S. J. (2018) 'The feasibility and acceptability of two methods of snack portion control in United Kingdom (UK) preschool children: Reduction and replacement', *Nutrients,* 10(10), pp. 1493. DOI: <https://doi.org/10.3390/nu10101493>.
13. Stevely, A. K., Buykx, P., Brown, J., Beard, E., Michie, S., Meier, P. S. and Holmes, J. (2018) 'Exposure to revised drinking guidelines and ‘COM-B’determinants of behaviour change: descriptive analysis of a monthly cross-sectional survey in England', *BMC Public Health,* 18(1), pp. 1-9. DOI: <https://doi.org/10.1186/s12889-018-5129-y>.
14. Stevens, K. (2012) 'Valuation of the child health utility 9D index', *PharmacoEconomics,* 30(8), pp. 729-747. DOI: <https://doi.org/10.2165/11599120-000000000-00000>.
15. West, J. H., Belvedere, L. M., Andreasen, R., Frandsen, C., Hall, P. C. and Crookston, B. T. (2017) 'Controlling Your "App"etite: How Diet and Nutrition-Related Mobile Apps Lead to Behavior Change', *JMIR mHealth and uHealth,* 5(7), pp. e95. DOI: <https://doi.org/10.2196/mhealth.7410>.
