# Supplementary material for: The impact of the Change4Life Food Scanner app on children’s diets and parental psychological outcomes: a randomised pilot and feasibility study
Source: BMC Public Health. 2025 Jul 2;25:2215. doi: 10.1186/s12889-025-23400-0 (PMC12220540; doi:10.1186/s12889-025-23400-0)
Supplement: Supplementary file 2 — Additional file 2 (docx). Demographics of study dropouts. A table outlining the characteristics of participants who did not complete the study. [file 12889_2025_23400_MOESM2_ESM.docx]

Additional File 2. Demographics of study dropouts ^a^

|  |  | **Total**  *n* = 62 | **Intervention**  *n* = 33 | **Control**  *n* = 29 |
| --- | --- | --- | --- | --- |
| **Child age (years)** ^b^ | Mean (±SD) | 6.67 (±1.85) | 6.78 (±1.53) | 6.56 (±2.15) |
| **Child sex** ^b^ | N (%) Female | 26 (48) | 13 (48) | 13 (48) |
|  | N (%) Male | 28 (52) | 14 (52) | 14 (52) |
| **Parent Ethnicity** ^c^ | N (%) White British | 39 (75) | 21 (81) | 18 (69) |
|  | N (%) White other | 3 (6) | 1 (3.8) | 2 (8) |
|  | N (%) Asian | 8 (15) | 2 (8) | 6 (23) |
|  | N (%) Mixed White and Black | 1 (2) | 1 (4) | 0 (0) |
|  | N (%) Other | 1 (2) | 1 (4) | 0 (0) |
| **Parent Education** ^c^ | N (%) Higher education ^d^ | 35 (67) | 18 (69) | 17 (65) |
|  | N (%) Other | 17 (33) | 8 (31) | 9 (35) |
| **Household Income (quintiles)** | N (%) Q1 – most deprived | 8 (13) | 5 (15) | 3 (10) |
|  | N (%) Q2 | 0 (0) | 0 (0) | 0 (0) |
|  | N (%) Q3 | 9 (15) | 3 (9) | 6 (21) |
|  | N (%) Q4 | 15 (24) | 9 (27) | 6 (21) |
|  | N (%) Q5 – least deprived | 17 (27) | 7 (21) | 10 (35) |
|  | N (%) Unknown | 13 (21) | 9 (27) | 4 (14) |
| N.B. Percentages rounded up to 0 decimal places. ^a^ Study dropouts defined as those who did not complete outcome measures, at baseline and/or 3 month follow up, after completing the study consent form.  ^b^ Missing cases: intervention = 6; control = 2  ^c^ Missing cases: intervention = 7; control = 3 ^d^ Defined as higher education qualification below degree level, degree level qualification, or a Masters/PhD or equivalent | | | | |
