# Supplementary material for: The impact of the Change4Life Food Scanner app on children’s diets and parental psychological outcomes: a randomised pilot and feasibility study
Source: BMC Public Health. 2025 Jul 2;25:2215. doi: 10.1186/s12889-025-23400-0 (PMC12220540; doi:10.1186/s12889-025-23400-0)
Supplement: Supplementary file 3 — Additional file 3 (docx). A Within-Subjects Comparison (Mean ±SD) of Psychological Predictors of Behaviour Change Between Baseline and 3-Month Follow-up. A table outlining mean differences between baseline and follow-up survey responses relating to psychological predictors of behaviour change. [file 12889_2025_23400_MOESM3_ESM.docx]

Additional File 3: A Within-Subjects Comparison (Mean ±SD) of Psychological Predictors of Behaviour Change Between Baseline and 3-Month Follow-up.

| **Measure** | **Intervention (*n=29*)** | | | **Control (*n=35*)** | | |
| --- | --- | --- | --- | --- | --- | --- |
|  | Baseline | 3MFU | Mean difference (95% CI) | Baseline | 3MFU | Mean difference (95% CI) |
| ***Attitudes*** | | | | | | |
| *How important is it for you that your family eat a healthy diet?* | 4.03 (±0.63) | 4.00 (±0.71) | -0.03  (-0.22; 0.16) | 4.17 (±0.62) | 4.03 (±0.57) | -0.14  (-3.66; 0.80) |
| *Having too much sugar leads to disease* | 4.28 (±0.75) | 4.48 (±0.634) | 0.21  (-0.07; 0.48) | 4.43 (±0.61) | 4.43 (±0.70) | 0.000  (-0.20; 0.20) |
| *When buying food, snacks or drinks for my child, it is important to pay attention to the amount of sugar it contains (attitudes)* | 4.21 (±0.77) | 4.17 (±0.76) | -0.03  (-0.31; 0.24) | 4.29 (±0.62) | 4.37 (±0.55) | 0.09  (-0.14; 0.31) |
| *For my child to be healthy, I need to be careful how much saturated fat my child eats* | 4.03 (±0.68) | 3.93 (±0.75) | -0.10  (-0.32; 0.11) | 4.11 (±0.72) | 4.17 (±0.79) | 0.06  (-0.13; 0.24) |
| *For my child to be healthy, I need to be careful how much sugar my child eats* | 4.55 (±0.51) | 4.38 (±0.72) | -0.17 (-0.40; 0.06) | 4.57 (±0.50) | 4.57 (±0.56) | 0.000  (-0.19; 0.19) |
| *For my child to be healthy, I need to be careful how many calories my child eats* | 3.28 (±0.88) | 3.10 (±1.11) | -0.17  (-0.58; 0.24) | 3.26 (±1.05) | 3.50 (±1.19) | 0.24  (-0.06; 0.53) |
| ***Perceived behavioural control*** ^a^ | | | | | | |
| *How much control do you have over your child’s sugar consumption?* | 3.89 (±0.74) | 3.75 (±0.70) | -0.14  (-0.42; 0.13) | 4.09 (±0.61) | 3.91 (±0.74) | -0.17  (-0.48; 0.14) |
| ***COM-B measures: Physical capability ^b^*** | | | | | | |
| *How often, if at all, do you keep track of how much sugar your child eats or drinks each day?* | 2.76 (±1.35) | 2.86 (±0.99) | 0.10  (-0.37; 0.57) | 2.74 (±1.38) | 3.00 (±1.18) | 0.27  (-0.05; 0.58) |
| ***COM-B measures: Psychological capability*** | | | | | | |
| *How easy or difficult do you find it to limit your child's sugar intake to the amounts recommended in the above guidelines?* | 3.07 (±1.39) | 3.10 (±1.54) | 0.03  (-0.73; 0.80) | 3.47 (±1.35) | 3.24 (±1.30) | -0.24  (-0.79; 0.32) |
| *“Too much sugar intake for my child increases their risk of obesity”* | 4.59 (±0.57) | 4.66 (±0.48) | 0.07  (-0.11; 0.24) | 4.69 (±0.47) | 4.63 (±0.55) | -0.06  (-0.26; 0.15) |
| ***COM-B measures: Automatic motivation*** | | | | | | |
| *How concerned, if at all, are you about your child consuming more sugar than what is recommended?* | 2.90 (±1.01) | 2.97 (±1.02) | 0.07  (-0.42; 0.56) | 3.15 (±0.89) | 3.03 (±0.94) | -0.12  (-0.53; 0.29) |
| *To what extent do you want to keep your child's sugar consumption within recommended guidelines?* | 3.69 (±0.85) | 3.79 (±1.01) | 0.10  (-0.25; 0.46) | 3.97 (±0.76) | 3.94 (±0.69) | -0.03  (-0.21; 0.15) |
| ***COM-B measures: Reflective motivation*** | | | | | | |
| *To what extent do you intend to keep your child's sugar consumption within recommended guidelines?* | 4.07 (±0.70) | 3.90 (±0.94) | -0.17  (-0.42; 0.08) | 4.24 (±0.70) | 4.18 (±0.72) | -0.06  (-0.27; 0.15) |
| *To what extent are you actively trying to reduce your child's sugar intake?* | 3.41 (±0.83) | 3.48 (±0.99) | 0.07  (-0.27; 0.41) | 3.59 (±0.74) | 3.44 (±0.79) | -0.15  (-0.42; 0.13) |
| ***COM-B measures: Social opportunity*** | | | | | | |
| *How easy or difficult do you think your lifestyle makes it for you to limit your child's sugar intake to the above guidelines, a day?* | 2.90 (±0.98) | 2.76 (±1.06) | -0.14  (-0.54; 0.27) | 3.41 (±0.99) | 3.47 (±0.90) | 0.06  (-0.28; 0.40) |
| N.B. Outcomes are based on 5-point Likert scales: 1 = negative attitudes (e.g. not at all important; none at all; strongly disagree; never; definitely not); 5 = positive attitudes (e.g. extremely important; a great deal; strongly agree; always; definitely yes). ^a^ intervention arm, n=28 ^b^ sample size of this measure onwards, control arm, n=34 | | | | | | |
