# Supplementary material for: The impact of the Change4Life Food Scanner app on children’s diets and parental psychological outcomes: a randomised pilot and feasibility study
Source: BMC Public Health. 2025 Jul 2;25:2215. doi: 10.1186/s12889-025-23400-0 (PMC12220540; doi:10.1186/s12889-025-23400-0)
Supplement: Supplementary file 4 — Additional file 4 (docx). Potential external influencers. An extension of the results section reporting baseline physical activity, awareness of external food policies and the impact of the coronavirus pandemic. [file 12889_2025_23400_MOESM4_ESM.docx]

**Additional File 4: Potential External Influencers**

*Physical activity*

Child physical activity was parent-reported at baseline. Those in the intervention condition (n=39) reported an average of 75 weekday minutes (±40.7), though this was subject to the removal of one outlier (600 minutes of typical weekday physical activity, 6SD above the mean), and 116 weekend minutes (±68.2) of physical activity. Those in the control arm (n=37) reported an average of 86 weekday (±53.4) and 107 weekend (±66.7) minutes of physical activity. Weekday and weekend days combined suggest similar physical activity levels between groups.

*Awareness of external food policies*

At 3MFU, participants were asked whether the introduction of the sugar tax led to changes in beverage purchasing behaviours; 64% of the sample (n=64) answered ‘never’ and 25% agreed that it had impacted on their behaviour to some extent. Similarly, when asked whether the introduction of the sugar tax reduced their child’s sugar intake, 66% of the sample responded ‘never’, whereas 25% reported a reduction in sugar intake to some extent. One respondent provided feedback at the end of the study that open-ended responses were needed to clarify choice of answers (“*I don't think the sugar tax questions were worded correctly because they didn't give a chance to explain the responses. Sugar was removed from some products to avoid the sugar tax (Ribena we are looking at you) and replaced with artificial sweeteners… so you could say that the sugar tax has influenced that behaviour but not in the way that the question was worded to measure*”).

When asked to report on their familiarity with the Change4Life campaign, 6% of the sample reported no familiarity, 50% of the sample reported some familiarity, and 44% reported high familiarity with the campaign. Despite this, 33% of the sample claimed to not currently use Change4Life resources, 52% sometimes use such resources, and 15% of the sample use Change4Life resources frequently. When asked whether existing public health campaigns and messages have helped to improve their child’s diet, 31% of the sample agreed to some extent, whereas 25% did not agree and 14% were not aware of any public health campaigns or messages.

*Impact of the Coronavirus pandemic*

As this study was disrupted by COVID-19, participants were asked a series of questions relating to the impacts of COVID-19 on their child’s diet and their participation in the study (see Supplementary Table 1). The majority of participants agreed that COVID-19 affected food purchasing behaviour (51%), led their child to eat more snacks than they did before (61%), eat more home cooked meals (76%), and spend more money on food (72%). Most participants disagreed that COVID-19 led to an increase in take-out food consumption (59%). When asked whether COVID-19, or any other events, affected responses or engagement in the trial, 48 (76.2%) of respondents answered no. When asked whether there were any other factors that may have had an influence over child sugar consumption in the last 3 months, 53 of 64 (83%) said no. For those that responded yes, open-ended responses were grouped into 3 themes. It was found that *lockdown demands caused time constraints*, whereby one participant reported, “life became hectic going back to work and home-schooling so had difficulty completing all tasks”, whilst another similarly said, “second survey was pandemic peak–- we struggled to fit in the surveys also”. Results also suggested that the pandemic had resulted in changes to individuals’ dietary behaviours (*changes to diet)*. One respondent reported, “only in the first few weeks of lockdown when I couldn’t buy my usual groceries.” Another respondent referred back to lockdown and school closures, “because at school her food intake would be very different”. Finally, being *out of routine* during the pandemic was found to affect engagement with the trial; one respondent reported, “being at home has increased snack consumption”.

| **Supplementary** Table 1. Impact of the COVID-19 Lockdown on Children’s Diets | | | | |
| --- | --- | --- | --- | --- |
| **Measure** | **n** | **High agreeability (%)** | **Medium agreeability (%)** | **Low agreeability (%)** |
| *To what extent do you feel that the lifestyle changes imposed by the Government in relation to the Coronavirus has affected the following*^a^*:* | | | | |
| COVID has affected your child’s diet | 55 | 29 | 42 | 29 |
| COVID has affected your ability to make healthier food choices for your child | 46 | 20 | 46 | 35 |
| COVID has affected your food purchasing behaviour | 55 | 51 | 44 | 6 |
| COVID has affected the types of food you bought | 46 | 37 | 50 | 13 |
| COVID has affected your participation in this study | 55 | 31 | 36 | 33 |
| COVID has affected your ability to scan barcodes using the Food Scanner app | 19 | 32 | 42 | 26 |
| Did the Food Scanner app support you at this time in making healthier food choices? | 19 | 26 | 37 | 37 |
| *“The lifestyle changes imposed by the Government in relation to the Coronavirus led my child to…”* ^b^ | | | | |
| …eat more sugar than they did before | 63 | 29 | 32 | 40 |
| …eat more snacks than they did before | 62 | 61 | 10 | 29 |
| …eat more fruit and vegetables than they did before | 62 | 44 | 39 | 18 |
| …eat more home cooked meals than they did before | 62 | 76 | 15 | 10 |
| … be more physically active than they were before | 62 | 40 | 24 | 36 |
| *To what extent do you feel that the lifestyle changes imposed by the Government in relation to the Coronavirus has affected the following, in comparison to before the lockdown* ^c^*:* | | | | |
| Since the COVID-19 lockdown, I carry out online grocery shopping… | 44 | 43 | 43 | 14 |
| Since the COVID-19 lockdown, my children eat take out food… | 46 | 9 | 33 | 59 |
| Since the COVID-19 lockdown, I have been purchasing sugary foods or treats/snacks… | 46 | 39 | 39 | 22 |
| Since the COVID-19 lockdown, I have been spending on food… | 46 | 72 | 17 | 11 |
| N.B. Questions pertaining to the Food Scanner app were only presented to those within the intervention condition.  Lower sample sizes than total number of study completers (n=64) was due to the late introduction of these measures.  ^a^ Response options: a great deal, a lot, a moderate amount, a little, not at all.  ^b^ Response options: strongly agree, somewhat agree, neither agree nor disagree, somewhat disagree, strongly disagree.  ^c^ Response options: A lot more, slightly more, the same, slightly less, a lot less. | | | | |
